# Strong Relationship between Oral Dose and Tenofovir Hair Levels in a Randomized Trial: Hair as a Potential Adherence Measure for Pre-Exposure Prophylaxis (PrEP)

**DOI:** 10.1371/journal.pone.0083736

**Published:** 2014-01-08

**Authors:** Albert Y. Liu, Qiyun Yang, Yong Huang, Peter Bacchetti, Peter L. Anderson, Chengshi Jin, Kathy Goggin, Kristefer Stojanovski, Robert Grant, Susan P. Buchbinder, Ruth M. Greenblatt, Monica Gandhi

**Affiliations:** 1 Bridge HIV, San Francisco Department of Public Health, San Francisco, California, United States of America; 2 Department of Medicine, University of California San Francisco (UCSF), San Francisco, California, United States of America; 3 Department of Bioengineering and Therapeutic Sciences, University of California San Francisco, San Francisco, California, United States of America; 4 Department of Epidemiology and Biostatistics, University of California San Francisco, San Francisco, San Francisco, California, United States of America; 5 Department of Pharmaceutical Sciences, University of Colorado, Aurora, Colorado, United States of America; 6 Children's Mercy Hospital and Clinics, University of Missouri-Kansas City, Kansas City, Missouri, United States of America; 7 Gladstone Institutes, San Francisco, California, United States of America; 8 Department of Clinical Pharmacy, University of California San Francisco, San Francisco, San Francisco, California, United States of America; Massachusetts General Hospital, United States of America

## Abstract

**Background:**

Pre-exposure prophylaxis (PrEP) trials using tenofovir-based regimens have demonstrated that high levels of adherence are required to evaluate efficacy; the incorporation of objective biomarkers of adherence in trial design has been essential to interpretation, given the inaccuracy of self-report. Antiretroviral measurements in scalp hair have been useful as a marker of long-term exposure in the HIV treatment setting, and hair samples are relatively easy and inexpensive to collect, transport, and store for analysis. To evaluate the relationship between dose and tenofovir concentrations in hair, we examined the dose proportionality of tenofovir in hair in healthy, HIV-uninfected adults.

**Methods:**

A phase I, crossover pharmacokinetic study was performed in 24 HIV-negative adults receiving directly-observed oral tenofovir tablets administered 2, 4, and 7 doses/week for 6 weeks, with a ≥3-week break between periods. Small samples of hair were collected after each six-week period and analyzed for tenofovir concentrations. Geometric-mean-ratios compared levels between each pair of dosing conditions. Intensive plasma pharmacokinetic studies were performed during the daily-dosing period to calculate areas-under-the-time-concentration curves (AUCs).

**Results:**

Over 90% of doses were observed per protocol. Median tenofovir concentrations in hair increased monotonically with dose. A log-linear relationship was seen between dose and hair levels, with an estimated 76% (95% CI 60–93%) increase in hair level per 2-fold dose increase. Tenofovir plasma AUCs modestly predicted drug concentrations in hair.

**Conclusions:**

This study found a strong linear relationship between frequency of dosing and tenofovir levels in scalp hair. The analysis of quantitative drug levels in hair has the potential to improve adherence measurement in the PrEP field and may be helpful in determining exposure thresholds for protection and explaining failures in PrEP trials. Hair measures for adherence monitoring may also facilitate adherence measurement in real-world settings and merit further investigation in upcoming PrEP implementation studies and programs.

**Trial Registration:**

ClinicalTrials.gov +NCT00903084.

## Introduction

Recent studies have provided new hope for effective HIV biomedical prevention strategies [1]–[4]. The efficacy of pre-exposure prophylaxis (PrEP), where at-risk HIV uninfected individuals take antiretroviral medications daily to prevent infection, has been demonstrated in several recent trials. The iPrEx study demonstrated that daily administration of emtricitabine/tenofovir disoproxil fumarate (FTC/TDF) PrEP decreased HIV-1 acquisition in men who have sex with men [2]. The Partners PrEP trial and Botswana TDF2 study subsequently verified and extended these findings in serodiscordant couples and seronegative heterosexual adults respectively [3], [4]. Most recently, a trial in HIV-uninfected injection drug users (IVDU) in Thailand showed a reduction in HIV incidence with the use of daily tenofovir [5]. Cumulative data from these trials led to the approval of FTC/TDF as PrEP for uninfected individuals at risk of HIV infection by the Food and Drug Administration [6] and/or the development of interim guidance for clinicians prescribing PrEP in different populations [7]–[9].

Importantly, several trials have highlighted the critical relationship between adherence and PrEP efficacy. For example, the efficacy of FTC/TDF in iPrEx rose from 44% overall to an estimated 92% among those with detectable blood drug levels [2]. Furthermore, two large PrEP trials in sexually active African women [10], [11] were unable to demonstrate significant efficacy of daily FTC/TDF in reducing HIV acquisition, likely due in large part to low adherence to study drug.

Incorporating measures of drug exposure as biomarkers of PrEP adherence has been critical to interpreting PrEP trials. In iPrEx, although mean adherence by self-report was 95%, drug was detected in only 8% of seroconverters compared with 54% of matched active-arm controls who remained uninfected [2]. Self-reported adherence was similarly high in both FEM-PrEP [10] and VOICE [11], but random plasma tenofovir (TFV) levels among women on active drug revealed target (≥10 nanograms per milliliter) or detectable TFV concentrations in only 26% and 29% of women, respectively. The limitations of self-report and other commonly used adherence measures in HIV treatment monitoring are well-described [12], and self-reported adherence may be particularly inaccurate in clinical trials [13]. Biomarkers of drug exposure can serve as surrogates of adherence because nonadherence is the most frequent cause of low drug levels. Pharmacologic parameters may be especially critical to monitor in HIV-negative individuals on antiretroviral prophylaxis since HIV viral loads cannot serve as indicators of adherence in noninfected persons.

Due to TFV's extended intracellular half-life (∼150 hours) [14], an ideal adherence biomarker would reflect average drug exposure over several weeks to months. Single plasma concentrations, which represent only a small window of exposure (dosing in the last few days) [15]–[17], are subject to significant day-to-day variation [15] and “white-coat” effects [18], [19] and are imperfect indicators of exposure. As the concentration of drugs in hair reflects uptake from the systemic circulation over an extended time window (weeks to months) [20], [21], hair analysis provides an advantage over plasma monitoring in assessing average drug exposure over a longer period of time. We have demonstrated that hair concentrations of antiretrovirals (ARVs) are the strongest independent predictor of virologic success in large prospective cohorts of HIV-infected patients [22]–[25]. Analyzing antiretroviral levels in hair may be a promising approach to objectively quantify adherence to PrEP regimens as well. Although the measurement of intracellular tenofovir diphosphate (TFV-DP) concentrations in peripheral blood mononuclear cells (PBMCs) has been incorporated into previous PrEP trials as a long-term measure of exposure, hair levels may provide feasibility advantages in the field since methods to process, isolate and count PBMCs are costly, technically challenging, and difficult to institute widely. Unlike phlebotomy, hair collection is noninvasive and does not require specific skills, refrigeration, or sterile equipment. Hair can be stored at room temperature prior to analysis and shipped without biohazard precautions.

To widely employ hair as a biological marker of drug exposure in HIV prevention settings, the relationship between different dosing strategies and drug concentrations in this matrix must be established. Moreover, individual variation in pharmacokinetic (PK) parameters (e.g. plasma oral clearance measured as areas-under-the-concentration-time-curve or AUCs) can contribute to variability of antiretroviral drug levels in various compartments [26], so the effect of these parameters on hair drug concentrations must be determined. We performed a phase I randomized crossover study with the objective of examining the impact of varying dosing patterns of oral TDF (2, 4 and 7 doses per week using directly observed dosing) and individual plasma AUCs on TFV concentrations in hair in healthy, HIV-negative adults. These dosing frequencies were chosen to represent low, moderate, and high adherence to PrEP. Furthermore, modeling studies suggest TFV-DP levels achieved with these dosing patterns were associated with increasing levels of PrEP efficacy [27]. In this study, we found a strong relationship between dose and hair levels of TFV, paving the way for further study of hair measures as an adherence monitoring tool in PrEP trials and implementation projects.

## Methods

### Study Design and Participant Selection

The protocol for this trial and supporting CONSORT checklist are available as supporting information; see Checklist S1 and Protocol S1.

STRAND was an open-label, randomized, three-period PK study conducted between October 2009 and March 2011 at the University of California San Francisco (UCSF) Clinical Research Center and the San Francisco Department of Public Health (SFDPH) Bridge HIV research clinic. To facilitate directly observed dosing, participants were recruited from UCSF (students, staff, and faculty) and from neighborhoods adjoining SFDPH. Tablets of TDF (300 milligrams, mg) were provided by Gilead Sciences (Foster City, CA). As several PrEP trials were evaluating TDF alone as a PrEP agent [3], [5], [28] and no significant pharmacokinetic interactions have been observed between tenofovir and emtricitabine [29], TDF tablets alone were chosen for evaluation to minimize potential toxicity to healthy subjects in this study. All subjects provided written informed consent prior to study procedures and the study was approved by the UCSF Committee on Human Research.

Eligible subjects were healthy male or female volunteers age 18 or older without serious or active medical conditions. Participants were all HIV-negative based on HIV rapid antibody testing and were hepatitis B surface antigen negative at enrollment. Enrolled participants could not be on nephrotoxic agents, had to have an estimated creatinine clearance ≥60 mL/min, serum creatinine levels below the upper limit of normal, urine dipstick tests that were negative or trace positive for glucose and protein, adequate hepatic and hematologic function, and negative urine pregnancy tests (for women). Female volunteers were required to use effective contraception during the study.

All participants had to be willing to undergo directly observed dosing (DOT), provide personal cell phone numbers for unobserved modified DOT (mDOT) visits, have dark (brown or black) hair (to minimize interindividual variability in this proof-of-concept study), a minimum occipital scalp hair length of 3 centimeters (cm), and not have used hair dyes or hair permanent products in the past 3 months. Participants at high risk of HIV infection, including those reporting injection drug use (IDU); vaginal or anal intercourse with an HIV-positive partner, IDU partner, or with more than 4 partners of the opposite sex; and/or transactional sex in the prior 6 months were excluded.

### Study Visit and Dosing Procedures

**Figure 1:**
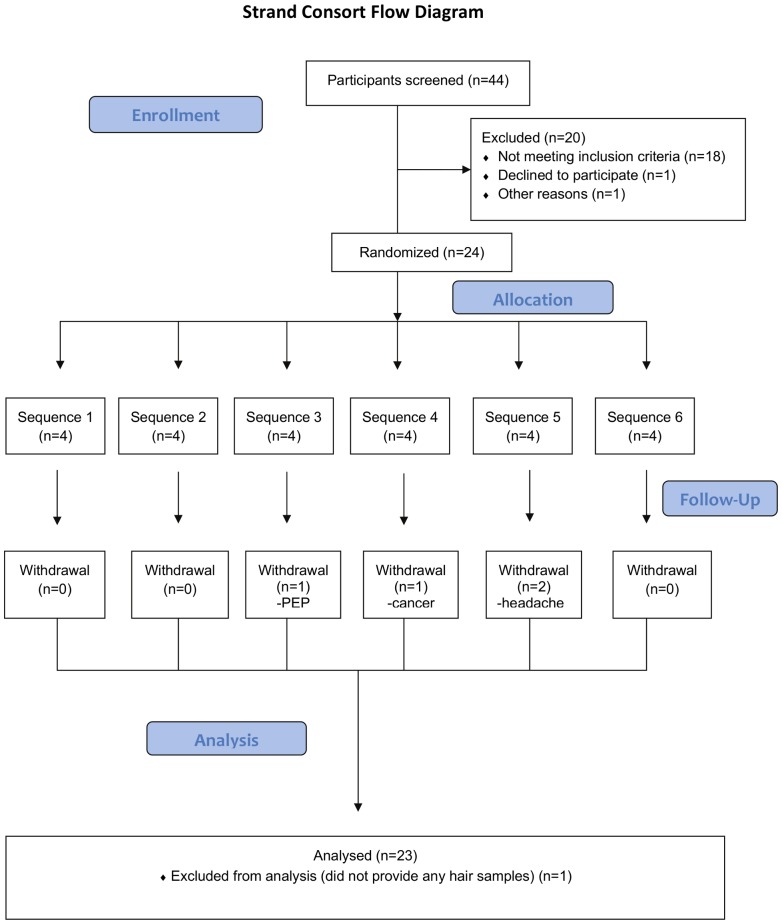
CONSORT flowchart for STRAND study.

**Figure 2:**
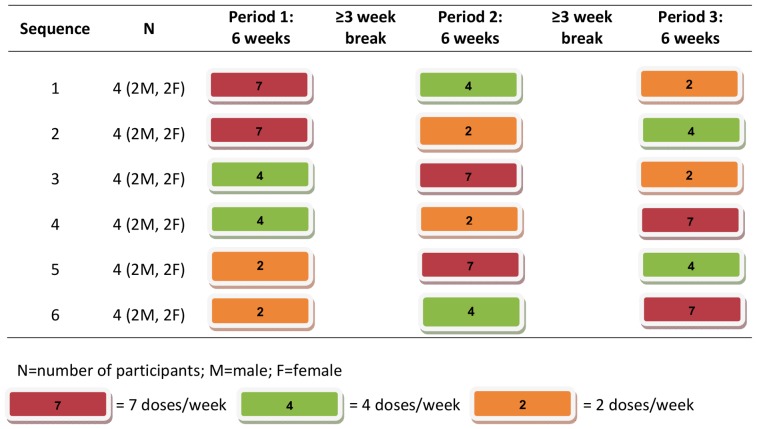
Dosing scheme for the STRAND Study.

Subjects were randomly assigned to one of 6 dosing sequences and completed three 6-week dosing periods of 2, 4 and 7 doses per week, with a break of at least 3 weeks between periods (**Figures 1** and **2**). Based on an average hair growth rate of 1 centimeter per month [30], a minimum of 3-weeks was chosen for the washout period. Each sequence arm included both sexes equally for a total of 12 male and 12 female participants. The 2 doses/week were taken on Tuesday and Wednesday, and the 4 doses/week on Monday, Tuesday, Thursday, and Friday. All scheduled doses on Monday to Friday were directly observed by study staff, and weekend doses were confirmed by text messaging or phone using previously-described mDOT procedures [31], [32]. A one-week supply of back-up medication was provided for participants unable to complete a directly-observed dose; these doses were confirmed via phone or text message at dosing. Dosing visits occurred at UCSF, SFDPH, or another location per participant convenience. Participants returned to the clinic for follow-up at a start, mid-point (after 3 weeks of dosing), and end-point (at the end of each 6 week dosing period) visit for safety and adverse event monitoring, symptom-directed physical exams, and rapid HIV testing.

At enrollment and end-point visits, approximately 150–200 strands of scalp hair were collected; additionally, a similar number pubic hair strands were collected on an opt-in basis. After 4 weeks of dosing during the 7 doses/week period (at steady state for plasma TFV), participants were admitted to the UCSF Clinical Research Center for intensive 24-hour plasma PK evaluations to calculate individual PK parameters (plasma AUC for oral clearance and C_max_). The participant's usual diet was ascertained prior to the PK visit, and simulation of the usual diet was undertaken during PK sampling. An initial blood level was drawn (called the “0” timepoint) prior to an observed dose of TFV, followed by blood collection at 0.25, 0.5, 1, 1.5, 2, 3, 4, 6, 8, 10, 12, 16, and 24 hours post-dose for measurement of TFV plasma concentration. Concentrations could not be measured for one participant at the 0.5 hour time and another participant at the 1.5 hour time; these were interpolated by fitting quadratic functions to the log-concentrations using the two previous and two subsequent times.

### Sample Collection and Processing

Several small thatches of hair (∼150–200 strands) were cut as close as possible to the scalp in the occipital region and/or pubic region, and the distal portion labeled [22]. The proximal section of the scalp or pubic hair sample (about 1.5 cm) was cut down and chopped to 1–2 mm length segments with scissors and 5 mg were weighed, processed and analyzed using liquid chromatography/tandem mass spectrometry (LC/MS-MS). The tenofovir in the cut hair sample was extracted with 50% methanol/water containing 1% trifluroacetic acid, 0.5% hydrazine dihydrochloride, and internal standard in a 37°C shaking water bath overnight (>12 hours) and then analyzed by a modified LC-MS/MS method [33]. The relative error (%) and precision (coefficients of variation (CV)) for spiked quality control hair samples at low, medium and high concentrations were all <15% (CV of 10.5%, 8.4%, and 6.0%, respectively). Each sample was analyzed once due to the limited number of clinical hair samples. The method to analyze TFV in hair was validated from 0.002 nanograms (ng)/mg to 0.400 ng/mg hair, with a lower limit of quantitation at 0.002 ng/mg [34], [35].

Plasma was collected in EDTA tubes for drug concentration measurement for the intensive pharmacokinetic studies. Plasma was analyzed for TFV via standard techniques of LC/MS/MS [36]. The linearity of the concentration curves is in the range of 10 to 1000 ng/milliliter (mL), and the lower limit of quantification is 10 ng/mL.

### Statistical Analyses

All analyses were conducted using SAS (Version 9.2, SAS Institute, Cary, NC) and Stata (Version 12.1, College Station, TX). The primary outcome variable was concentration of TFV in hair at each end-point visit. Median drug levels with 95% confidence intervals (CIs) were calculated, and geometric mean ratios with 95% CIs were used to compare levels between each pair of dosing conditions. We used geometric means to avoid overweighting large ratios and to match the logarithmic transformation used for the multivariate modeling. Spaghetti plots were used to depict drug levels for each participant, with each line representing an individual. The chosen sample size (n = 24) was not based on formal power considerations, but was feasible and expected to provide useful new information about typical relationships between TDF dosing and drug concentrations, as well as a preliminary assessment of how well hair levels could distinguish between daily versus intermittent dosing.

Linear mixed models with random intercepts were used for multivariable analyses of predictors of logarithmically transformed hair levels. Plasma AUC_0–24_, a measure of the subject's oral clearance (dose*F/CL), was calculated using the linear-log trapezoidal method, and C_max_ was defined as the largest observed TFV concentration in plasma over the 24 hours. The primary predictor variables were dosing condition (2, 4, and 7 dose/week), log-transformed plasma AUC, and log-transformed C_max_. We examined linearity assumptions for continuous predictors by adding quadratic terms to the models.

## Results

### Sociodemographics, Safety, and Dosing:

Twenty-four participants met eligibility criteria and were enrolled (**Figure 1**). Demographics and baseline data for the 23 participants who provided one or more hair samples are shown in **Table 1**.

**Table 1:** Demographics of participants who provided hair samples (n = 23).

Variable	N	%
**Sex**		
Female	12	52%
Male	11	48%
**Race/Ethnicity**		
Non-Hispanic White	12	52%
Asian/Pacific Islander	3	13%
Latino/Hispanic	7	30%
Other	1	4%
**Education**		
Some college	9	39%
Bachelors	9	39%
Graduate	5	22%

Variable	N	Mean	SD	IQR
**Age (in years)**	23	34.2	9.0	26.6–41.1
**Creatinine Clearance** *	23	129.4	31.1	107.2–154.6
**Weight (in kg)**	23	73.9	13.2	64.1–86.3

Estimated by the Cockcroft-Gault equation [57].

N = number of participants; SD = standard deviation; kg = kilograms. IQR = interquartile range.

There were 167 adverse events (AEs) reported during the study, with each participant reporting at least 1 AE. Most AEs were either mild, grade 1 (128 events, 76%) or moderate, grade 2 (37 events, 22%). The most commonly reported AEs were headache (54%), upper respiratory infection (38%), nausea (29%), abdominal gas (17%), soft/loose stools (17%), and fatigue (17%). Only one participant experienced a grade 3 AE (traumatic jaw fracture) and one experienced a grade 4 AE (metastatic breast cancer), both adjudicated as unrelated to study drug. Four participants withdrew before completing all study procedures: one after the first TDF dose due to a grade 2 hypersensitivity reaction (urticarial rash with tongue tingling) occurring prior to PK data collection, one before completing the 4 and 7 doses/week periods due to headaches, one before completing the 2 doses/week period due to use of post-exposure prophylaxis and elevated pancreatic enzymes, and one before completing the 7 doses/week period due to the new cancer diagnosis described above.

Over 90% of doses were observed according to protocol: 249/267 (93%) doses in the 2 doses/week arm, 473/517 (91%) doses in the 4 doses/week arm, and 773/856 (90%) in the 7 doses/week arm. Of these 1495 doses observed on protocol, 82% were DOT and 18% were mDOT visits. An additional 130 (7.9%) doses were taken from participants' back-up medication supplies (confirmed via phone/text message). Two participants were unable to attend several consecutive DOT visits during their 7 doses/week period due to travel; all 31 of these doses were confirmed on time via phone or text. Combining per protocol doses and confirmed doses from back-up supplies, 1624/1640 (99%) of expected doses were taken according to prescribed schedules Overall, 55/67 (82%) of dosing periods had 100% on-time completion rates, and only 2 periods had <90% completion: one participant missed 3/12 (25%) doses during the 2 doses/week period, and another participant missed 4/36 (11%) doses in the 4 doses/week period.

### TFV concentrations in hair

Median TFV levels in scalp hair increased monotonically from 2 to 4 to 7 doses/week (**Table 2**). There was minimal overlap in TFV hair concentrations between 2 versus 7 doses/week, with some overlap between the 2 versus 4 and 4 versus 7 doses/week dosing periods. The geometric mean ratios were close to the ratios of the doses: 4 versus 2 doses/week averaged 1.76 (versus 2.0 ratio of doses); 7 versus 4 doses/week averaged 1.60 (versus 1.75 ratio of doses); 7 versus 2 doses/week averaged 2.85 (versus 3.5 ratio of doses).

**Table 2:** Median drug levels and geometric mean ratios in hair after 6 weeks of use, by dosing period.

	Hair (scalp)	
Dosing period	N	Median (range) TFV levels (ng/mg)
2 doses/week	22	0.012 (0.008 to 0.021)
4 doses/week	22	0.023 (0.011 to 0.042)
7 doses/week	21	0.038 (0.021 to 0.053)

Period comparison		Geometric mean ratios (95% CI)
4 vs. 2 doses/week	21	1.76 (1.45 to 2.13)
7 vs. 4 doses/week	21	1.60 (1.34 to 1.90)
7 vs. 2 doses/week	20	2.85 (2.46 to 3.32)

N = number; TFV = tenofovir; ng/mg = nanogram/milligram; CI = confidence interval.

**Figure 3:**
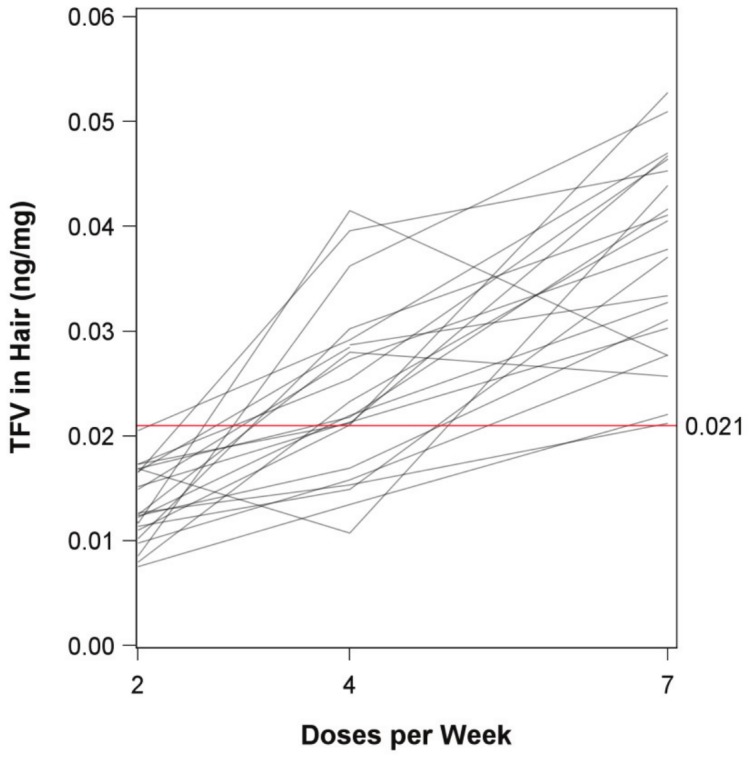
Spaghetti plot of tenofovir (TFV) concentrations. Hair TFV concentrations for each subject are shown for each dosing period. Each line represents drug concentration data from one participant (at 2, 4, and 7 doses/week). The red line distinguishes between hair scalp concentrations for 2 vs. 7 doses/week. ng/mg = nanogram/milligram.

**Figure 3** depicts the scalp hair TFV concentrations for the 2, 4, and 7 doses/week periods for each individual. A concentration of TFV in hair of 0.021 ng/mg reliably distinguished between 2 versus 7 doses/week (before rounding, all levels for 2 doses/week were <0.021 ng/mg, and all levels for 7 doses/week were >0.021 ng/mg). Tenofovir hair concentrations were higher for 7 versus 4 doses/week and 4 versus 2 doses/week in all but 3 participants (**Table 3**). These 3 participants did not have fewer observed doses or other signs of altered adherence. Coefficients of variation were 0.27 for hair at 2 doses/week, 0.34 at 4 doses/week, and 0.25 at 7 doses/week.

**Table 3:** Three participants where hair levels did not demonstrate monotonic increases.

Patient ID	Hair TFV (ng/mg) at 2 doses/week	Hair TFV (ng/mg) at 4 doses/week	Hair TFV (ng/mg)at 7 doses/week
039	0.017	0.011	0.044
051	0.013	0.028	0.026
063	0.012	0.042	0.028

ID = identification number; TFV = tenofovir; ng/mg = nanogram per milligram.

In a model with log(doses per week) and log(AUC) as predictors, a log-linear relationship was seen between doses per week and TFV scalp hair level, with an estimated 76% (95% CI 60–93%, p<0.0001) increase in hair level per 2-fold dose increase. Adding a random dose effect to this model estimated minimal inter-individual variability in dose effect (estimated random effect variance of zero), suggesting very similar dose effects across most subjects. TFV plasma AUCs, ranging from 1210 to 5610 ng×h/mL, were a moderate predictor of TFV hair level, with a 23% increase in hair level per 2-fold increase in AUC (p = 0.035). Allowing for different residual variance at different dose levels (heteroscedasticity) resulted in similar dose and AUC estimates. Also, similar results were observed when restricting analyses to the 7 doses/week period where intensive PK parameters were measured (27% increase in hair level per 2-fold increase in AUC, p = 0.042). C_max_ appeared to have little positive effect on hair level when controlled for dose and AUC (−20% per 2-fold increase, 95% CI −43% to +10%, p = 0.16). There was no statistically significant effect of total body weight (p = 0.35), estimated creatinine clearance (p = 0.52), or sex (p = 0.89) on TFV hair concentrations.

Pubic hair TFV levels varied erratically and did not monotonically increase in 5/11 subjects who had at least two on-drug pubic hair samples. In addition, levels were exceedingly high in 6 samples (∼30–300 times higher than TFV levels in scalp hair).

## Discussion

Accurate adherence measures are critical to the interpretation of PrEP clinical trials and may play an important role in monitoring PrEP use in clinical practice. An effective pharmacologic measure of PrEP adherence in a particular matrix must demonstrate a clear relationship between dose and drug levels. Our analysis provides evidence of a proportional relationship between dose and hair levels of TFV in small samples of scalp hair from healthy, HIV-uninfected individuals. Specifically, there was an estimated 76% increase in TFV hair level with doubling of dose, with similar dose effects across subjects. Hair concentrations exhibited linearity with dose in the range of 2 to 7 doses per week, with minimal inter-individual variation in dose effect. A hair concentration of 0.021 ng/mg distinguished between 2 versus 7 doses/week dosing. This discrimination ability may have useful clinical applications in monitoring PrEP use, as a previous PK modeling study demonstrated that estimated protective efficacy for taking FTC/TDF 2 doses/week was 76% (95% CI 56 to 96%), compared with a projected 99% (95% CI 96 to >99%) for 7 doses/week [27]. There were 3/23 participants in which TFV dose was not monotonically increasing. In all 3 cases, these discrepancies were between 2 vs. 4 doses/week or 4 vs. 7 doses/week.

Pubic hair samples were not useful in measuring TFV concentrations and varied erratically in this study, with several high outlier values. Similar results have been reported for pubic hair levels of opiates [37], amphetamines [38], benzodiazepines [39], and rodenticidal toxins [40], and may reflect urinary contamination of pubic hair or the slower growth rate of pubic hair. Furthermore, individual PK parameters, including oral clearance as measured by plasma AUC, and C_max_, had modest predictive value on drug levels in hair. The limited predictive value of PK parameters in this study may have been due to within-person variation over time in PK parameters (such that a single PK measure may not completely represent systemic drug exposure over the entire study period), or other factors that may influence hair levels, such as differences in hair growth rate and drug in sweat that came in contact with sampled hair [41].

Antiretroviral drug levels in hair have been shown to be highly correlated with treatment outcomes in HIV-infected patients, demonstrating stronger predictive value than self-reported adherence [22], [24] or single plasma ARV concentrations [23], [24]. Similarly, objective biological markers of PrEP adherence have been used to predict protective efficacy in PrEP trials [2]–[4]. While qualitative drug detection overall has demonstrated utility in predicting PrEP efficacy across a number of PrEP studies [42], quantitative drug levels may provide further value in predicting protective effects and could also be used in establishing potential threshold drug levels for protection. For example, a regression analysis in iPrEx estimated that a tenofovir-diphosphate (TFV-DP) concentration of 16 femtomole (fmol)/10^6^ PBMCs was associated with a 90% reduction in HIV acquisition [27]. Similarly, women in the CAPRISA study of 1% vaginal tenofovir gel who had a tenofovir concentration of >1000 ng/mL in cervicovaginal fluid samples had a significant reduction in HIV acquisition compared with those on placebo, in contrast to women with tenofovir concentrations of ≤1000 mg/mL, who did not [43]. Quantitative drug levels can also be used to understand PrEP failures. In the randomized phase of the iPrEx study, TFV-DP concentrations in peripheral blood mononuclear cells was detected in only 3/42 HIV seroconverters at the visit with first evidence of HIV infection, and quantitative TFV-DP levels were low (and below the range expected for daily dosing) in these individuals at this time point [27]. Finally, quantitative drug levels could be tested in real-time or near-time and be used to identify individuals who may need additional adherence support while taking PrEP in upcoming PrEP studies and demonstration projects. For example, having an undetectable or low TFV hair drug level after PrEP initiation could trigger further evaluation of patterns of adherence and/or potential individual PK parameters affecting TFV clearance. Patients with evidence of low adherence could receive a more intensive adherence intervention and more frequent drug monitoring in PrEP implementation programs. Important next steps for the use of hair as a biological marker of PrEP exposure will be to establish the acceptability of hair collection in real-world PrEP delivery settings, determine the relationship between hair and PrEP efficacy in upcoming PrEP studies, assess the impact of different dosing patterns (e.g. occasional missed doses vs. longer drug holidays) on hair TFV levels, and evaluate the use of hair levels in interpreting PrEP breakthrough infections. These data will help determine whether analysis of hair drug levels could be used as a primary monitoring tool of adherence in upcoming PrEP studies and programs.

As tenofovir's long intracellular half life could afford TDF-based PrEP regimens some PK forgiveness [44], the protective effect of PrEP is more likely to be affected by longer treatment interruptions rather than occasional missed doses, a scenario similar to missed doses to non-nucleoside reverse transcriptase inhibitor (NNRTI)-based regimens in HIV-infected individuals with low to moderate adherence [45]. Strategies to improve the utility of hair analyses to detect drug interruptions, such as segmental hair analysis (analysis of short, contiguous segments of hair from the scalp end) [46], warrant further investigation. Given the consistent growth rate of hair in the occipital region of the scalp at approximately 1 centimeter/month, analysis of shorter segments of hair (e.g. proximal 0.5 cm of hair growth) may be useful in monitoring brief periods of PrEP use (e.g. preceding 2 weeks of PrEP use).

Our study had several strengths, including use of a randomized cross-over study design, high adherence to mDOT procedures ensuring dosing fidelity, and intensive PK sampling to evaluate key PK parameters that could influence drug concentrations in various compartments. This study also had several limitations. First, participation was limited to individuals with dark hair, as we aimed to reduce inter-individual variability and maximize the ability of hair to discriminate between different adherence patterns in this proof-of-concept study. While this may limit the generalizability of our findings, many PrEP studies are conducted in international settings where most individuals have dark hair. Second, oral clearance at steady state was only measured once during the 7 doses/week period to reduce participant burden, which did not allow for assessment of intra-subject variability at the other dosing regimens. However, the predictive value of plasma AUC on tenofovir hair levels did not substantially improve in analyses predicting hair levels only after the 7 doses/week period. Third, weekend or holiday doses were not directly observed due to feasibility issues; however, these doses were confirmed by text message or phone. This mDOT approach has been demonstrated to be effective in prior studies [31], [47]. Fourth, likely due to our limited recruitment venues to facilitate DOT dosing, we did not enroll African-American participants into this proof-of-concept study. As African-Americans are disproportionately impacted by HIV [48] and are likely be a prioritized group for PrEP, future studies evaluating the PK of TFV and FTC in hair should include this important population. Another limitation is that hair samples were only analyzed once, due to the limited quantity of hair collected from subjects. Finally, this study measured drug levels after 6 weeks of dosing, which may not represent “steady state” concentrations in the proximal 1.5 cm of hair due to growth rate variability. Additional studies are needed to evaluate whether TFV levels in proximal hair samples continue to increase with longer dosing periods (e.g. 3–6 months).

In summary, this study reports a consistent relationship between tenofovir dose and hair in HIV-negative men and women. These measures have already demonstrated utility for other ARVs in the HIV treatment setting and the finding of a strong dose relationship for tenofovir in hair paves the way for evaluation of its use in monitoring adherence in tenofovir-based PrEP demonstration projects and real-world PrEP settings. Unlike phlebotomy, hair collection is inexpensive, noninvasive and does not require specific skills, sterile equipment, or specialized storage conditions. While this hair PK study collected 150–200 strands of hair, ∼100 strands are typically needed for TFV level analysis. Hair can be stored for prolonged durations prior to analysis at room temperature and shipped without biohazardous precautions or cold chain requirements. These features may make this monitoring tool particularly promising in resource-poor settings, where high acceptability of hair collection has been demonstrated [49]–[51]. While the current equipment used for the LC-MS/MS method of hair analysis is expensive, methods to analyze hair using thin layer chromatography (TLC), a simple and inexpensive analytical tool that has been used to detect ARVs in human plasma, saliva, and umbilical cord blood [52]–[54] are currently being developed or have been developed [55] and may be performed by local laboratories in resource-poor settings. A low-cost, point-of-care hair assay [55] could also facilitate real or near-time testing of hair samples, and results could be used to inform PrEP adherence counseling.

Quantitative, time-integrated measures of long-term drug exposure in hair may provide more salient adherence information than qualitative (e.g. detectable/undetectable) results or drug levels in single plasma samples. Furthermore, our results may be useful in interpreting data from intermittent PrEP trials. Such dose reduction strategies are expected to lower costs and toxicity, but would be difficult to evaluate without validated biomarkers of adherence. Given that adherence is the “Achilles' heel” of PrEP [56], this novel biomarker examining drug levels in hair has the potential to improve adherence measurement in PrEP trials, implementation studies, PrEP adherence promotion strategies, and real-world use.

## Supporting Information

Checklist S1 **CONSORT Checklist.**
(DOCX)

Protocol S1 **Trial Protocol.**
(DOCX)
