# Supplementary material for: Strong Relationship between Oral Dose and Tenofovir Hair Levels in a Randomized Trial: Hair as a Potential Adherence Measure for Pre-Exposure Prophylaxis (PrEP)
Source: PLoS One. 2014 Jan 8;9(1):e83736. doi: 10.1371/journal.pone.0083736 (PMC3885443; doi:10.1371/journal.pone.0083736)
Supplement: Protocol S1 — Trial Protocol. (DOCX) [file pone.0083736.s002.docx]

**A Phase 1 Pharmacokinetic Study of**

**Varying Dosing Patterns on Tenofovir Concentrations in Hair**

**Sponsored by:**

**National Institutes of Mental Health**

**NCT# 00903084**

**Protocol Chair:
Albert Liu, MD, MPH**

**Version 6.0**

**September 23, 2010List of Abbreviations and Acronyms**

AE adverse event

ALT alanine transaminase

ART antiretroviral therapy

AST aspartate aminotransferase

AUC area under the curve

BMD bone mineral density

BUA Biological use authorization

C Celsius

CA California

CBC complete blood count

CDC Center for Disease Control and Prevention

CFR Code of Federal Regulations

CHR Committee on Human Research

CL/F oral clearance

cm centimeter

C_max_ maximum serum concentrations

C_min_ minimum serum concentrations

CRC Clinical Resource Center

CRF case report form

D4T stavudine

DAIDS Division of AIDS

dL deciliter

DNA deoxyribonucleic acid

EDTA ethylenediaminetetraacetic acid

EFV efavirenz

EIA Enzyme Linked Immunosorbent Assay

F Fahrenheit or female

FDA (United States) Food and Drug Administration

FHI Family Health International

FTC emtricitabine

HBsAg Hepatitis B surface antigen

HCG human chorionic gonadotropin

HIV Human Immunodeficiency Virus

HIV-1 Human Immunodeficiency Virus-1

ID identification

IDU injection drug user

IFA immunofluorescent assay

IRB Institutional Review Board

IUD intrauterine device

kg kilogram

mDOT modified directly observed therapy

mg milligram

mL milliliter

min minute

M male

MSM men who have sex with men

MTF male to female
N number (sample size)

NIAID National Institute of Allergy and Infectious Disease

NIH National Institutes of Health

NIMH National Institutes of Mental Health

NRTI nucleoside reverse transcriptase inhibitor

NtRTI nucleotide reverse transcriptase inhibitor

OHRP Office for Human Research Protections

PBMC peripheral blood mononuclear cell(s)

PE physical exam

PI protease inhibitor

PK pharmacokinetic(s)

pm post-meridiem (after noon)

PMPA 9-[2-(Phosphonomethoxy)propyl]adenine

PrEP pre-exposure prophylaxis

RA research associate

RT reverse transcriptase

SAE serious adverse event

SFDPH San Francisco Department of Public Health

TDF tenofovir disoproxil fumarate

TFV tenofovir

TFV-DP tenofovir diphosphate

t_max_ time to maximum concentration

UA urinalysis

UCSF University of California, San Francisco

ULN upper limits of normal

UNAIDS Joint United Nations Programme on HIV/AIDS

US United States

USA United States of America

UW University of Washington

WB Western blot

Wks weeks

3TC lamivudine

**TABLE OF CONTENTS**

[1 PROTOCOL TEAM ROSTER 6](#_Toc260239767)

[2 PROTOCOL SUMMARY 9](#_Toc260239768)

[3 INTRODUCTION 11](#_Toc260239769)

[3.1 Background 11](#_Toc260239770)

[3.2 Tenofovir Disoproxil Fumarate (TDF) 14](#_Toc260239771)

[3.2.1 Description 14](#_Toc260239772)

[3.2.2 Strength of the Study Product 15](#_Toc260239773)

[3.2.3 Preclinical Pharmacology and Toxicology 15](#_Toc260239774)

[3.2.4 Clinical Studies 16](#_Toc260239775)

[3.2.5 Pharmacokinetics 16](#_Toc260239776)

[3.2.6 Safety 17](#_Toc260239777)

[3.2.7 Safety Results from a Completed PrEP Trial 20](#_Toc260239778)

[4 STUDY OBJECTIVES 21](#_Toc260239779)

[4.1 Primary Objectives 21](#_Toc260239780)

[4.2 Exploratory Objectives 21](#_Toc260239781)

[5 STUDY DESIGN 21](#_Toc260239782)

[5.1 Expected Duration of Enrollment and Subject Participation 21](#_Toc260239783)

[5.2 Description of Study Population 22](#_Toc260239784)

[5.3 Inclusion Criteria 22](#_Toc260239785)

[5.4 Exclusion Criteria 23](#_Toc260239786)

[5.5 Recruitment 25](#_Toc260239787)

[6 STUDY PRODUCT 26](#_Toc260239788)

[6.1 Formulation 26](#_Toc260239789)

[6.2 Dispensing and Dosing 26](#_Toc260239790)

[6.3 Supply 27](#_Toc260239791)

[6.4 Accountability 27](#_Toc260239792)

[7 STUDY PROCEDURES 27](#_Toc260239793)

[7.1 Study Flow Chart 27](#_Toc260239794)

[7.2 Drug-Administration Flowchart 28](#_Toc260239795)

[7.3 Screening 28](#_Toc260239796)

[7.4 Enrollment 30](#_Toc260239797)

[7.5 Allocation Scheme 31](#_Toc260239798)

[7.6 Randomization 31](#_Toc260239799)

[7.7 Follow-up Visits 32](#_Toc260239800)

[7.8 Directly Observed Visits and Phone Visits 32](#_Toc260239801)

[7.9 Interim Contacts 33](#_Toc260239802)

[7.10 Criteria for Suspension of Study Product 33](#_Toc260239803)

[7.11 Follow-up Procedures for Participants who Discontinue Study Product 34](#_Toc260239804)

[7.12 Early Study Termination Visit 34](#_Toc260239805)

[8 DATA SOURCES 35](#_Toc260239806)

[8.1 Study Eligibility Assessment 35](#_Toc260239807)

[8.2 Clinical Assessment 35](#_Toc260239808)

[8.3 Toxicity Monitoring 36](#_Toc260239809)

[8.4 Monitoring for Pregnancy 36](#_Toc260239810)

[8.5 HIV-1 Testing 36](#_Toc260239811)

[8.6 Hair Specimens 36](#_Toc260239812)

[8.7 Intensive PK Specimens 37](#_Toc260239813)

[8.8 PBMC Specimens 37](#_Toc260239814)

[8.9 HIV-1 Cultures of PBMCs in vitro 38](#_Toc260239815)

[8.10 Additional Cryopreservation of Blood Specimens 39](#_Toc260239816)

[9 ADVERSE EVENTS 39](#_Toc260239817)

[9.1 Assessment of Adverse Events 39](#_Toc260239818)

[9.2 Serious Adverse Event 40](#_Toc260239819)

[9.3 Serious Adverse Event Reporting Requirements 40](#_Toc260239820)

[9.4 Recording of Clinical and Laboratory AEs 41](#_Toc260239821)

[9.5 Adverse Event Relationship to Study Product 41](#_Toc260239822)

[9.6 Grading Severity of Adverse Events 42](#_Toc260239823)

[9.7 Toxicity Management 42](#_Toc260239824)

[9.7.1 Creatinine Elevations 43](#_Toc260239825)

[9.8 Management of Intercurrent Medications 44](#_Toc260239826)

[9.9 HIV and Hepatitis B 44](#_Toc260239827)

[9.10 Clinical Management of Pregnancy 44](#_Toc260239828)

[9.11 Social AEs 44](#_Toc260239829)

[9.12 Review of AEs 45](#_Toc260239830)

[10 DATA MANAGEMENT AND STATISTICAL CONSIDERATIONS 45](#_Toc260239831)

[10.1 Study Primary Endpoint and Predictor Variables 45](#_Toc260239832)

[10.2 Study Hypotheses 46](#_Toc260239833)

[10.3 Biostatistical Methods 46](#_Toc260239834)

[10.4 Sample Size 48](#_Toc260239835)

[10.5 Data Management 48](#_Toc260239836)

[11 HUMAN SUBJECTS CONSIDERATIONS 49](#_Toc260239837)

[11.1 Institutional Review Boards 49](#_Toc260239838)

[11.2 Informed Consent 49](#_Toc260239839)

[11.3 Participant Confidentiality 50](#_Toc260239840)

[11.4 Risks 50](#_Toc260239841)

[11.5 Benefits 51](#_Toc260239842)

[11.6 Access to Clinician 51](#_Toc260239843)

[11.7 Special Populations 51](#_Toc260239844)

[11.7.1 Pregnant Women 51](#_Toc260239845)

[11.7.2 Children 52](#_Toc260239846)

[11.8 Incentives 52](#_Toc260239847)

[11.9 Treatment for Injury 52](#_Toc260239848)

[11.10 Data and Safety Monitoring 52](#_Toc260239849)

[11.11 Study Discontinuation 53](#_Toc260239850)

[11.12 Biohazard Containment 53](#_Toc260239851)

[12 ADMINISTRATIVE PROCEDURES 54](#_Toc260239852)

[13 REFERENCES 55](#_Toc260239853)

[14 LIST OF APPENDICES 57](#_Toc260239854)

# PROTOCOL TEAM ROSTER

**Protocol Chair**

**Albert Liu, MD, MPH**

**Protocol Chair**

San Francisco Department of Public Health

25 Van Ness Avenue, Suite 500

San Francisco, CA 94102

T: 415-554-9104

F: 415-621-0641

[albert.liu@sfdph.org](mailto:albert.liu@sfdph.org)

**National Institute of Mental Health (NIMH)**

**Andrew D. Forsyth Ph.D.**

**Project Officer**

National Institute of Mental Health

6001 Executive Blvd, MSC 9619

Bethesda MD 20892

T: 301-443-8403

F: 301-443-9719

aforsyth@mail.nih.gov

**Gilead Sciences**

**James F. Rooney, MD**

**Vice President Clinical Research**

Gilead Sciences

333 Lakeside Dr.

Foster City, CA 94404 USA

T: 650-522-5708

F: 650-522-5854

Jim.rooney@gilead.com

**University of California, San Francisco (UCSF)**

**Ruth Greenblatt, MD**

**Co-Investigator**

405 Irving Street, Second Floor

San Francisco, CA 94122

T: 415-502-6287

F: 415-476-8528

[ruth.greenblatt@ucsf.edu](mailto:ruth.greenblatt@ucsf.edu)

**Monica Gandhi, MD**

**Co-Investigator**

405 Irving Street, Second Floor

San Francisco, CA 94122

T: 415-502-6285

F: 415-476-8528

[Monica.gandhi@ucsf.edu](mailto:Monica.gandhi@ucsf.edu)

**Joseph B. Guglielmo, PharmD**

**Co-Investigator**

521 Parnassus Avenue 152

San Francisco, CA 94143

T: 415-476-2352

F: 415-476-6632

[guglielmo@pharmacy.ucsf.edu](mailto:guglielmo@pharmacy.ucsf.edu)

**Yong Huang, Ph.D.**

**Co-Investigator**

296 Lawrence Street

San Francisco, CA 94143

T: 415-476-5220

F: 415-476-6770

[yong.huang@ucsf.edu](mailto:yong.huang@ucsf.edu)

**Peter Bacchetti, Ph.D.**

**Co-Investigator**

185 Berry Street 5700

San Francisco, CA 94143-0560

T: 415-514-8030

F: 415-514-8150

[peter@biostat.ucsf.edu](mailto:peter@biostat.ucsf.edu)

**Gladstone Institute of Virology and Immunology**

**Robert, Grant, MD, MPH**

**Co-Investigator**

1650 Owens Street

San Francisco, CA 94158

T: 415-734-4810

F: 415-355-0855

[robert.grant@ucsf.edu](mailto:robert.grant@ucsf.edu)

**University of Missouri-Kansas City**

**Kathleen J. Goggin, Ph.D.**

**Co-Investigator**

4825 Troost Street, Suite 111-D

San Francisco, CA 64110-2499

T: 816-235-1059

F: 816-235-1062

[goggink@umkc.edu](mailto:goggink@umkc.edu)

**San Francisco Department of Public Health**

**Susan Buchbinder, MD**

**Co-Investigator**

25 Van Ness Avenue, Suite 710

San Francisco, CA 94102

T: 415-554-9070

F: 415-431-7029

[susan.buchbinder@sfdph.org](mailto:susan.buchbinder@sfdph.org)

**Allison Futeral**

**Study Coordinator**

San Francisco Department of Public Health

25 Van Ness Avenue, Suite 500

San Francisco, CA 94102

T: 415-554-8456

F: 415-621-0641

# [allison.futeral@sfdph.org](mailto:allison.futeral@sfdph.org)

#

# PROTOCOL SUMMARY

**Protocol title:** Phase 1 Pharmacokinetic Study of Varying Dosing Patterns on Tenofovir Hair Concentrations

**Clinical Phase:** 1

**Protocol Chair:** Albert Liu, MD, MPH

**Sample Size:** 24

**Trial Site:** University of California, San Francisco; San Francisco Department of Public Health

**Study Population:** Healthy, HIV-uninfected men and women age 18 or older

**Study Design:** Phase 1, open-label, randomized, six sequence, three period, single-site cross-over study

**Study Duration:**  Approximately 20-36 weeks per participant (18 weeks on study drug)

**Study Regimen:**

| **Sequence** | **N** | **Period A:**  **6 weeks** | **Break** | **Period B:**  **6 weeks** | **Break** | **Period C:**  **6 weeks** |
| --- | --- | --- | --- | --- | --- | --- |
| 1 | 4 (2M, 2F) | 7 doses/week |  | 4 doses/week |  | 2 doses/week |
| 2 | 4 (2M, 2F) | 7 doses/week |  | 2 doses/week |  | 4 doses/week |
| 3 | 4 (2M, 2F) | 4 doses/week |  | 7 doses/week |  | 2 doses/week |
| 4 | 4 (2M, 2F) | 4 doses/week |  | 2 doses/week |  | 7 doses/week |
| 5 | 4 (2M, 2F) | 2 doses/week |  | 7 doses/week |  | 4 doses/week |
| 6 | 4 (2M, 2F) | 2 doses/week |  | 4 doses/week |  | 7 doses/week |

**Primary Objectives:**

- To determine the impact of varying dosing patterns on tenofovir concentrations in hair among HIV-negative men and women under conditions of modified directly observed therapy
- To determine how individual pharmacokinetic parameters influence tenofovir hair concentrations and modify the relationship between dose and tenofovir hair concentrations.

**Exploratory objectives**

- To determine the correlation between intracellular tenofovir diphosphate concentrations and tenofovir levels in hair under varying dosing patterns
- To determine dose and intracellular concentrations of tenofovir sufficient to render cells non-permissive for HIV-1 replication in vitro
- To determine the correlation between TFV scalp hair and pubic hair concentrations

**Primary Endpoint:**

- **Tenofovir hair concentration.** Tenofovir hair levels will be determined using hair specimens collected at enrollment and on the last day of each dosing period. Absolute hair levels and ratios of hair levels between conditions will be determined.

**Exploratory Endpoints:**

- **Intracellular tenofovir concentrations.** The correlation between intracellular tenofovir-diphosphate concentrations and tenofovir hair concentrations on the last day of each dosing period will be determined.
- **HIV replication in vitro.** Dose and intracellular concentrations of tenofovir sufficient to render cells non-permissive for HIV-1 replication in vitro will be evaluated.
- **Pubic hair tenofovir concentrations:** TFV levels in scalp and pubic hair will be correlated under various dosing conditions.

# INTRODUCTION

## Background

UNAIDS estimates there are over 7,300 new HIV infections per day worldwide despite widespread knowledge of the protective effects of abstinence, monogamy, and condoms.^1^ Although behavior change has likely led to substantial reductions in HIV incidence and will remain the cornerstone of HIV prevention, new HIV prevention strategies are urgently needed to further reduce incident infections. Pre-exposure prophylaxis (PrEP), or the initiation of antiretroviral medication prior to potential HIV exposure, is a promising but unproven HIV prevention strategy that could have a significant impact on HIV transmission globally.^2-6^ Several PrEP trials are underway or in the planning stages to evaluate the safety and efficacy of tenofovir (TFV) or emtricitabine/TFV in diverse populations globally.

The accurate measurement of drug exposure to TFV, a component of all PrEP regimens, is critical for interpreting the primary scientific outcomes of PrEP studies. One of the primary goals of the current PrEP studies is to determine whether steady state levels of PrEP drugs confer protection from HIV infection in HIV-negative individuals. As for all medications, antiretroviral drugs must be present at the sites of action in appropriate concentrations to be effective.^7^ In individuals who seroconvert despite taking active study drug in PrEP efficacy trials, the key question will be whether these failures are due to a true lack of PrEP efficacy versus inadequate drug exposure. There is currently no gold standard to measure drug exposure in PrEP trials. Current PrEP trials are utilizing self-reported adherence measures, pill counts, and electronic data monitoring to assess medication adherence. However, each of these tools has significant limitations. In addition, adherence is an imperfect surrogate to assess total drug exposure since it does not account for interindividual biologic variability in exposure.

There is great interest in developing a biomarker of adherence and drug exposure in the PrEP field. HIV viral loads serve as a clear marker of response to therapy in HIV therapeutics, but the PrEP prevention field lacks an analogous surrogate for protection from HIV transmission. In the case of PrEP efficacy, it has been hypothesized that there may be a certain exposure threshold to TFV that is required to achieve protection from HIV infection. A reliable biomarker of drug exposure could therefore be used to determine an exposure threshold that correlates with protection. Future applications of this method include identifying individuals using PrEP that fall below this threshold to target additional adherence interventions, or in the case of optimal adherence, investigate biologic factors that contribute to suboptimal drug exposure.

An ideal biomarker of PrEP exposure would be noninvasive, inexpensive, precise, and not based on individual self-report or provider observation.

Due to TDF’s extended plasma (17 hours) and intracellular (>60 hours) half-life, an ideal biomarker would reflect average drug exposure over an extended period of time (similar to hemoglobin A1C as a surrogate for overall glycemic control in diabetes mellitus^8, 9^). Therefore, single blood levels, which provide only a brief snapshot of time, are imperfect indicators of exposure. Because drug is incorporated from blood into hair over weeks to months, hair has emerged as a highly promising biomarker of drug exposure over extended periods of time.^10, 11^ Hair is an excellent matrix for drug deposition, because it lacks drug metabolizing enzymes, and hair drug concentrations occur in proportion to blood concentrations.^12-15^ Variability in drug level measurements in hair can be minimized by collection of the hair sample from a consistent location.^16, 17^ Hair matrix preserves drug, allowing for room temperature storage over prolonged periods (months to years)^14^, facilitating its utility in domestic and international settings.^10^ Previous studies have demonstrated that hair levels of protease inhibitors (PIs) strongly correlate with virologic suppression in patients initiating a new PI-based regimen.^18^ More recently, methods for the measurement of TFV concentrations in hair have been developed and are now able to accurately determine whether individuals have been exposed to this drug.

In a pilot study of 68 participants enrolled in the CDC-sponsored tenofovir PrEP trial, we evaluated the feasibility of hair collection and established the range of TDF levels seen in hair collected from the occipital scalp. Of 74 participants approached, 92% agreed to hair collection. Only 1 participant in the placebo arm had a detectable TDF level at 0.0118 ng/mg. All patients in the TDF arm had detectable TDF levels in hair (median 0.0483 ng/mg) with levels spanning a dynamic range (0.0115 to 0.1060 ng/mg) (see figures 3.1.1 and 3.1.2).

**Figure 3.1.1 Figure 3.1.2**

Although these results are promising, the use of this assay is limited by the lack of understanding of what these levels mean and whether they are an adequate reflection of TFV drug exposure. Additional work is therefore required to further validate the hair assay before use as a biomarker for prophylactic TFV exposure in large PrEP efficacy trials. Because drug exposure is a reflection of both dosing (patient adherence) and individual PK parameters, demonstrating a correlation of dosing patterns and PK parameters to TFV hair levels would strongly suggest that hair is a reliable and accurate measure of drug exposure over time. The proposed study will address this gap and provide data to support hair collection and analysis in upcoming PrEP efficacy trials.

An open label study using modified directly observed therapy (mDOT) provides an ideal experimental design to evaluate the effect of varying dosing patterns and individual PK parameters on hair TFV concentrations. All current PrEP trials are randomized, double-blinded, and placebo-controlled trials using prescriptions for daily oral dosing. Therefore, embedding a study to evaluate the effect of varying dosing patterns within a larger PrEP trial is complicated by the potential risk of inadvertent premature unblinding and could adversely impact the goal of maximizing PrEP adherence in clinical trial participants. Furthermore, prescriptions in the trial for daily dosing create social desirability bias in reported adherence. Conducting this experiment in HIV-positive cohorts is complicated by the use of other antiretrovirals and concomitant medications which could influence plasma and hair levels of TFV and the importance of a high level of adherence in these patients. To simulate conditions most realistically in the PrEP trial setting, this study would need to be performed in HIV-noninfected individuals.

We therefore plan to conduct a 24 week open-label study of healthy HIV-uninfected volunteers to evaluate the effects of varying dosing patterns on TFV levels in hair. We will recruit students, faculty, and staff from an academic medical center who will likely understand the importance of PK studies and can be readily accessed in a central location. In this proof-of-concept study, we will restrict enrollment to individuals with untreated, naturally dark (brown or black) hair to reduce between-person variability, making hair levels more effective at distinguishing different adherence levels and patterns. Since many PrEP trials are taking place in developing countries where individuals have dark hair, our results will be applicable to studies in these countries as well as in many international treatment settings. This study utilizes a cross-over design in which each individual undergoes three 6 week conditions in random order: 7 doses/week (100% adherence), 4 doses/week (57% adherence), and 2 doses/week (29% adherence). These levels are chosen to represent excellent adherence, moderate adherence, and poor adherence, respectively. We will employ modified directly observed therapy (mDOT), an adherence intervention shown to be effective in improving virologic and immunologic outcomes in HIV treatment studies.^19-21^ This method will provide a high level of certainty that drug is actually being taken by the study population. The relatively short duration of this study and careful safety monitoring of this trial will minimize the risk of toxicity among study volunteers.

## Tenofovir Disoproxil Fumarate (TDF)

### Description

Tenofovir disoproxil fumarate is currently approved under the trade name Viread® for the treatment of HIV-1 infection in adults.^22^ TDF is the oral pro-drug of tenofovir, an acyclic nucleotide analog (9‑[(R)‑2‑(phosphonomethoxy)propyl]adenine monohydrate, PMPA) with activity in vitro against retroviruses, including HIV-1 and HIV-2, as well as hepadnaviruses. Tenofovir is metabolized intracellularly to tenofovir diphosphate (PMPApp), which is a competitive inhibitor of HIV‑1 reverse transcriptase (RT) that terminates the growing DNA chain. TDF is orally bioavailable in animals and humans and is rapidly converted to tenofovir on absorption. Further information regarding TDF, its toxicology, efficacy and safety profile will be reviewed in the text below and is available in the Viread® Package Insert (Appendix 1).

### Strength of the Study Product

The strength of the TDF tablets will be the dose approved by the FDA for the indication of treatment of HIV-1 infection in adults. For the treatment of HIV-1 infection, TDF is administered at 300 mg per day and has excellent activity against wild type and many drug-resistant viruses.

### Preclinical Pharmacology and Toxicology

Tenofovir and tenofovir disoproxil fumarate administered in toxicology studies to rats, dogs, and monkeys at exposures (based on AUCs) greater than or equal to 6-fold those observed in humans caused bone toxicity. In monkeys, the bone toxicity was diagnosed as osteomalacia. Osteomalacia observed in monkeys appeared to be reversible upon dose reduction or discontinuation of tenofovir. In rats and dogs, the bone toxicity manifested as reduced bone mineral density. The mechanism(s) underlying bone toxicity is unknown.

Evidence of renal toxicity was noted in 4 animal species. Increases in serum creatinine, BUN, glycosuria, proteinuria, phosphaturia, and/or calciuria and decreases in serum phosphate were observed to varying degrees in these animals.

Long-term oral carcinogenicity studies of TDF in mice and rates were carried out at exposures up to approximately 16 times (mice) and 5 times (rats) those observed in humans at the therapeutic dose for HIV-1 infection. At the high dose in female mice, liver adenomas were increased at exposures 16 times that in humans. In rats, the study was negative for carcinogenic findings at exposures up to 5 times that observed in humans at the therapeutic dose.

TDF was mutagenic in the in vitro mouse lymphoma assay and negative in an in vitro bacterial mutagenicity test (Ames test). In an in vivo mouse micronucleus assay, TDF was negative when administered to male mice.

There were no effects on fertility, mating performance or early embryonic development when TDF was administered to male rates at a dose equivalent to 10 times the human dose based on body surface area comparisons for 28 days prior to mating and to female rats for 15 days prior to mating through day seven of gestation. There was, however, an alteration of the estrous cycle in female rats. Reproductive studies have been performed in rats and rabbits at doses up to 14 and 19 times the human dose based on body surface area comparisons and revealed no evidence of impaired fertility or harm to the fetus due to tenofovir (Appendix 1).

### Clinical Studies

The US Food and Drug Administration (FDA) approved Viread® (TDF) in October 2001 for use in combination with other antiretroviral therapeutic (ART) agents for the treatment of HIV‑1 infection. In August 2008, TDF was approved by the FDA for the treatment of chronic hepatitis B in adults. Over 12,000 patients have participated in clinical trials or expanded access studies involving TDF for periods of 28 days to 215 weeks. A total of 1,544 patients have received TDF 300 mg once daily in clinical trials, and over 11,000 patients have received TDF in expanded access studies. TDF has been shown to be generally safe, effective, and well tolerated (Appendix 1).

### Pharmacokinetics

Tenofovir disoproxil fumarate is an oral prodrug of the active ingredient, tenofovir. The oral bioavailability of tenofovir from TDF in fasted patients is approximately 25%. TDF is eliminated by both active tubular secretion and glomerular filtration, and the TDF serum half‑life of ~17 hours and intracellular half-life of >60 hours supports a once-daily dosing schedule. Following oral administration of 300mg of TDF to HIV-infected patients in the fasted state, maximum serum concentrations are achieved in 1.0 ± 0.4 hours. Maximum serum concentration and AUC values are 296 ± 90 ng/ml and 2287 ± 685 ng·hr/mL, respectively. The pharmacokinetics of tenofovir are dose proportional over a TDF dose range of 75 to 600 mg and are not affected by repeated dosing. Tenofovir pharmacokinetics are similar in male and female patients. Significant drug interactions have been reported with coadministration of TDF with didanosine, atazanavir, and lopinavir/ritonavir. (Appendix 1).

### Safety

Numerous clinical studies have demonstrated TDF to have an excellent safety profile. Assessment of adverse reactions has been primarily based on studies among HIV-infected persons in which treatment-experienced and naïve patients received TDF or placebo as part of their ART regimens for at least 48 weeks. The most common adverse reactions (incidence ≥ 10%, Grades 2-4) identified from 3 large controlled clinical trials of TDF (903, 907, 934) include rash, diarrhea, headache, pain, depression, asthenia, and nausea.

Gilead Study 903, a randomized, double-blind trial conducted in the United States, Europe, and South America, was designed to compare the efficacy and safety of a treatment regimen of TDF, lamivudine (3TC), and efavirenz to a regimen of stavudine (d4T), lamivudine, and efavirenz in 600 antiretroviral-naïve HIV-1 infected patients through 144 weeks. The table below lists the grade 2-4 adverse events reported by at least 5% of patients in study 903, by study arm.

**Grade 2-4 Adverse Events Reported in > 5% patients in Study 903 (0-144 weeks)**

| **Type of Event** | **TDF+3TC+EFV**  **(N=299)** | **D4T+3TC+EFV**  **(N=301)** |
| --- | --- | --- |
| **General** |  |  |
| Headache | 14 % | 17% |
| Pain | 13 % | 12 % |
| Fever | 8% | 7% |
| Abdominal pain | 7% | 12% |
| Back pain | 9% | 8% |
| Asthenia | 6% | 7% |
| **Gastrointestinal** |  |  |
| Nausea | 8% | 9% |
| Diarrhea | 8% | 9% |
| Vomiting | 5% | 9% |
| Dyspepsia | 4% | 5% |
| **Metabolic Disorders** |  |  |
| Lipodystrophy | 1% | 8% |
| **Musculoskeletal** |  |  |
| Arthralgia | 5% | 7% |
| Myalgia | 3% | 5% |
| **Nervous system** |  |  |
| Depression | 11% | 10% |
| Insomnia | 5% | 8% |
| Dizziness | 3% | 6% |
| Peripheral neuropathy | 1% | 5% |
| Anxiety | 6% | 6% |
| **Respiratory** |  |  |
| Pneumonia | 5% | 5% |
| **Dermatologic** |  |  |
| Rash | 18% | 12% |

With the exception of fasting cholesterol and fasting triglyceride elevations that were more common in the stavudine group (40% and 9%) compared with TDF (19% and 1%) respectively, laboratory abnormalities observed in this study occurred with similar frequency in the TDF and stavudine treatment arms. Decreases in bone mineral density (BMD) were seen at the lumbar spine and hip in both study arms. At week 144, there was a significantly greater mean percentage decrease from baseline in BMD at the lumbar spine in patients receiving TDF + lamivudine + efavirenz (-2.2% ± 3.9) compared with patients receiving stavudine + lamivudine + efavirenz (-1.0% ± 4.6). Changes in BMD at the hip were similar between the two treatment groups. In both groups, the majority of the reduction in BMD occurred in the first 24-48 weeks of the study and this reduction was sustained through week 144. Twenty-eight percent of TDF-treated patients vs. 21% of the stavudine-treated patients lost at least 5% of BMD at the spine or 7% of BMD at the hip. Clinically relevant fractures (excluding fingers and toes) were reported in 4 patients in the TDF group and 6 patients in the stavudine group. There were significant increases in biochemical markers of bone metabolism (serum bone specific alkaline phosphatase, serum osteocalcin, serum C-telopeptide, and urinary N-telopeptide), suggesting increased bone turnover. Serum parathyroid hormone levels and 1,25 Vitamin D levels were also higher in the TDF group. Except for bone specific alkaline phosphatase, these changes resulted in values that remained within normal range. The effects of TDF-associated changes in BMD and biochemical markers on long-term bone health and future fracture risk are unknown.

Through 144 weeks, the renal safety profile was similar between the 2 groups. Two patients in each group developed a creatinine level of more than 2.0 mg/dL, while hypophosphatemia (<2.0 mg/dL) was observed in 10 patients receiving TDF and 8 patients receiving stavudine. The incidence of proteinuria and/or glucosuria was similar between the two groups. No patient developed Fanconi’s syndrome or discontinued study participation due to TDF-related renal abnormalities.

In Gilead Study 934, 511 antiretroviral-naïve patients received either TDF+FTC administered in combination with efavirenz (N=257) or zidovudine/lamivudine administered in combination with efavirenz (N=254). Adverse reactions and laboratory abnormalities observed in this study were generally consistent with those seen in previous studies in treatment experienced or treatment-naïve patients.

In Gilead Study 907, 550 treatment-experienced HIV patients in North America, Europe, and Australia were randomized to receive TDF or placebo in addition to their existing antiretroviral therapy. After 24 weeks of blinded, placebo-controlled dosing, all patients were switched to receive open-label TDF for the remaining 24 weeks. The adverse reactions seen in treatment experienced patients were generally consistent with those seen in treatment naïve patients including mild to moderate gastrointestinal events, such as nausea, diarrhea, vomiting and flatulence. Less than 1% of patients discontinued study participation in the clinical studies due to gastrointestinal adverse reactions (Appendix 1).

Information from post-marketing experience has noted the following adverse events which may be associated with TDF: common cold symptoms, allergic reactions (including angioedema), hepatotoxicity, and osteopenia which may infrequently lead to fractures.

### Safety Results from a Completed PrEP Trial

Peterson et al. conducted a randomized placebo controlled clinical trial of daily oral TDF 300mg versus placebo for HIV prevention in West African women. Nine-hundred thirty six HIV-negative women at high risk of HIV infection were observed for 428 person years for safety outcomes including monitoring of liver function tests, renal function, and clinical adverse events. No significant differences were evident between treatment groups in clinical or laboratory safety outcomes. Among the 368 participants on TDF, none had grade 3 or 4 transaminase elevation or grade 2 or higher creatinine. One TDF recipient had self-limited grade 3 decreased phosphate. Two HIV infections were diagnosed in participants randomized to TDF (0.86 per 100 person-years) and six in participants receiving placebo (2.48 per 100 person-years), yielding a rate ratio of 0.35 (95% confidence interval = 0.03-1.93), which did not achieve statistical significance. This study provides important and reassuring information on the safety of TDF chemoprophylaxis in HIV-uninfected women.

Several additional studies examining the safety and efficacy of oral tenofovir or emtricitabine/tenofovir in diverse populations are underway or in the later stages of planning.

These are shown in the table 3.2.7.

**Table 3.2.7 PrEP clinical trials currently planned or underway**

| **Sponsor** | **Product** | **Location** | **N** | **Study population** | **End date** |
| --- | --- | --- | --- | --- | --- |
| CDC | Oral TDF | Thailand | 2400 | Male/female IDU | 2010 |
| CDC | Oral TDF | USA | 400 | MSM | 2009 |
| CDC | Oral FTC/TDF | Botswana | 2000 | Heterosexual men & women | 2010 |
| NIH | Oral FTC/TDF | Andes, US, other sites | 3000 | MSM | 2010 |
| NIH | Vaginal TFV  Oral TDF  Oral FTC/TDF | Africa | 4200 | Women | 2012 |
| FHI | Oral FTC/TDF | Africa | 3900 | Women | 2012 |
| UW | Oral TDF  Oral FTC/TDF | Africa | 3900 couples | Serodiscordant couples | 2011 |

# STUDY OBJECTIVES

## Primary Objectives

- To determine the impact of varying dosing patterns on tenofovir concentrations in hair among HIV-negative men and women under conditions of modified directly observed therapy
- To determine how individual pharmacokinetic parameters influence tenofovir hair concentrations and modify the relationship between dose and tenofovir hair concentrations.

## Exploratory Objectives

- To determine the correlation between intracellular tenofovir diphosphate concentrations and tenofovir levels in hair under varying dosing patterns
- To determine dose and intracellular concentrations of tenofovir sufficient to render cells non-permissive for HIV-1 replication in vitro
- To determine the correlation between TFV scalp hair and pubic hair concentrations

# STUDY DESIGN

This is a phase 1, open label cross-over study to evaluate the impact of varying dosing patterns on concentrations of TFV in hair among a cohort of 24 HIV-uninfected men and women. In addition, the study will determine how individual PK parameters at steady state impact TFV hair concentrations.

## Expected Duration of Enrollment and Subject Participation

The approximate time to complete study enrollment is expected to be -12 months. Study participants will be followed over a 5-9 month period. The time of total study duration is expected to be approximately 6-10 months.

## Description of Study Population

The study population will include 24 generally healthy HIV-negative men and women age 18 or older. This study will plan to enroll 12 men and 12 women to provide PK data in both biologic sexes.

## Inclusion Criteria

Men and women must meet all of the following criteria to be eligible for inclusion in the study:

- Age 18 or older
- Willing and able to provide written informed consent
- Ability to speak English
- HIV-1 uninfected based on HIV rapid testing performed during screening and enrollment
- Calculated creatinine clearance ≥ 60 mL/min by the Cockcroft-Gault Creatinine Clearance Formula where creatinine clearance in mL/min is calculated as follows:

Male: [(140 - age in years) x (wt in kg)] / [72 x (serum creatinine in mg/dL)]
Female: [(140 - age in years) x (wt in kg) x 0.85] / [72 x (serum creatinine in mg/dL)]

- Serum creatinine less than or equal to the site laboratory upper limit of normal (ULN)
- Urine dipstick with negative or trace result for both glucose and protein
- Negative urine ß-HCG test (for women)
- Adequate hepatic function (total bilirubin and hepatic transaminases (ALT and AST) ≤ 2x ULN)
- Adequate hematologic function (absolute neutrophil count ≥ 1,500/mm^3^, platelets count ≥ 100,000/mm^3^, and hemoglobin ≥ 10 g/dL)
- Ability to participate in modified directly observed dosing of study drug
- Able to provide a personal cell phone number to be contacted on for unobserved mDOT visits
- Minimum length of 3 cm scalp hair in occipital region
- Willing to provide hair and plasma samples per protocol
- Dark hair (brown or black) assessed by the study clinician.
- Reproductive status: A volunteer who was born female must:
  - Agree to consistently use effective contraception from at least 21 days prior to enrollment through the last protocol visit for sexual activity that could lead to pregnancy. Effective contraception is defined as using any of the following methods:
    - Condoms (male or female) with or without a spermicide
    - Diaphragm or cervical cap with spermicide
    - Intrauterine device (IUD)
    - Hormonal contraception, or
    - Successful vasectomy in the male partner (considered successful if a volunteer reports that a male partner has [1] documentation of azoospermia by microscopy, or [2] a vasectomy more than 2 years ago with no resultant pregnancy despite sexual activity post-vasectomy);
  - Or not be of reproductive potential, such as having reached menopause (no menses for 1 year) or having undergone hysterectomy, bilateral oophorectomy, or tubal ligation
  - Or be sexually abstinent.
- Volunteers who were born female must also agree not to seek pregnancy through alternative methods, such as artificial insemination or in vitro fertilization until after the last scheduled protocol visit

## Exclusion Criteria

- Active and serious medical problems including cardiac or pulmonary disease, diabetes requiring hypoglycemia medications, previously diagnosed malignancies expected to require further treatment, and serious infections
- Hepatitis B surface antigen positivity or detectable Hepatitis B DNA in individuals who have isolated Hepatitis B core antibody positivity (HBSAg negative, anti-HBs negative, anti-HBc positive)
- History of chronic renal disease
- Known osteoporosis, osteomalacia, or osteopenia; history of pathological bone fractures not related to trauma
- Receiving ongoing therapy with any of the following: antiretroviral therapy, interferon or interleukin therapy, aminoglycoside antibiotics, amphotericin B, cidofovir, systemic chemotherapeutic agents, other agents with significant nephrotoxic potential, other agents that may inhibit or compete for elimination via active renal tubular secretion (e.g., probenecid), and/or other investigational agents
- Evidence of a gastrointestinal malabsorption syndrome or chronic nausea or vomiting which may confer an inability to receive an orally administered medication
- Use of hair dyes or hair permanent products in the last 3 months (streaking is acceptable)
- Current participation in any other research study involving drugs, investigational agents, or medical devices
- Per participant report, breastfeeding at screening or enrollment
- Active alcohol or drug use considered sufficient by clinician to hinder compliance with study procedures
- Elevated risk of HIV infection, including:

***Anyone*** *who has had one or more of the following risk factors* ***within the past 6 months****:*

- Used IV drugs recreationally
- Had vaginal or anal intercourse with HIV-infected partner(s);
- Had vaginal or anal intercourse with IDU partner(s) who injected drugs in the last 6 months
- Had syphilis, gonorrhea, non-gonoccocal urethritis, chlamydia, and/or pelvic inflammatory disease diagnosed or treated, based on self-report
- Had vaginal or anal intercourse with more than four partners of the opposite sex; and/or
- Exchanged sex for money, drugs, services, or gifts

***Man or male-to-female (MTF) transgender*** *who* ***within the past 6 months*** *has:*

- Had unprotected anal intercourse with 2 or more (male or transgender) sexual partners;
- Had anal intercourse with 4 or more male or MTF partners; and/or
- Used methamphetamines or cocaine at the time of anal intercourse with a male or MTF partner
- At enrollment, has any social or medical condition that, in the investigator’s opinion, would preclude provision of informed consent, make participation in the study unsafe, complicate interpretation of study outcome data, or otherwise interfere with achieving the study objectives

## Recruitment

Potential study volunteers will be primarily recruited through the UCSF campus community (students, staff, and faculty), as well as members of the general public. Members of the UCSF community can be easily reached for directly observed dosing of study drug. Study visits will occur on the UCSF Parnassus campus, at the San Francisco Health Department, or another location according to feasibility and convenience for the participants. Recruitment materials will be approved by the UCSF Institutional Review Board. Information about this study will be posted to a variety of UCSF electronic email listservs to assist with study recruitment. Additional recruitment methods may include advertising through local newspapers, using direct outreach, and through the Internet.

Interested subjects will be pre-screened over the phone or in person. If preliminarily eligible, they will be scheduled for an in-person screening visit in a private location at UCSF or at the SFDPH research clinic.

# STUDY PRODUCT

## Formulation

Tenofovir disoproxil fumarate (Viread®, TDF) oral tablet, is a fumaric acid salt of bisisopropoxycarbonyloxymethyl ester derivative of tenofovir. Each tablet contains 300 mg of TDF, which is equivalent to 245 mg of tenofovir disoproxil. Tenofovir disoproxil fumarate should be stored at 25ºC (77ºF). Excursions are permitted between 15ºC and 30ºC (59ºF and 86ºF)

## Dispensing and Dosing

Study participants will receive study drug using modified directly observed therapy (mDOT) conducted by a research associate (RA) trained in mDOT procedures. Study product will be dispensed to enrolled participants, upon receipt of a written prescription from an authorized prescriber. Participants will be randomized to one of six study regimen sequences (see section 7.5). Each participant’s study sequence will consist of three dosing periods of 6 weeks each, with a break of at least 3 weeks between dosing periods. For UCSF students, each study period will occur during an academic quarter, with a break between dosing periods of several weeks corresponding to the vacation breaks between quarters. The total study duration will be approximately five or more months for non-students, and approximately 36 weeks for UCSF students. Participants will take pills according to the dosing schedule designated by their randomized sequence (see section 7.5 for details) and meet with study staff for mDOT as described below (see section 7.8). Dosing times will be recorded by the study staff for the directly observed and telephone mDOT doses in a dosing log. Participants will be provided a one-week pre-packed emergency supply of medication to be used if they cannot or fail to make a scheduled visit with the study staff. Study drug may be taken without regard to meals.

## Supply

Tenofovir disoproxil fumarate 300 mg tablets will be supplied by Gilead Sciences, Inc. (Foster City, CA, USA). Tablets will be packaged in bottles of 30 tablets with a child-resistant screw cap. In addition to the tablets, each bottle contains a silica gel dessicant to protect the product from humidity and polyester packing material that cushions it during handling and shipping. Gilead will prepare bottles of study agent that are each labeled with expiry date and bottle ID number, which will be assigned to participants at the time of randomization during the enrollment visit.

## Accountability

Study drug will be stored at the San Francisco Department of Public Health HIV Research Section study site in a secured location in double-locked cabinets. The investigator and designated staff will maintain complete records of all study products received from Gilead and subsequently dispensed. At the end of the study, a final drug accounting of unused study material will be performed on proper log documents. All unused study product will either be returned to Gilead or destroyed according to site-approved procedures.

# STUDY PROCEDURES

## Study Flow Chart

| **Procedure** | **SCR** | **ENR** | **D1** | **MIDPT** | **ENDPT** |
| --- | --- | --- | --- | --- | --- |
|  |  |  |  |  |  |
| Informed Consent | X |  |  |  |  |
| Baseline Medical History | X |  |  |  |  |
| Full Physical Exam |  | X |  |  |  |
| Sx-directed Physical Exam | X |  | X | X | X |
| Adverse Event Assessment |  |  |  | X | X |
| Adherence counseling |  |  | X | X |  |
| CBC, chemistries | X | X | X | X | X |
| Urine dipstick | X | X | X | X | X |
| Urine pregnancy test (women only) | X | X | X | X | X |
| HBSAg, anti-HBs, anti-HBc† | X |  |  |  | X |
| HIV-1 rapid test | X | X | X | X | X |
| Hair collection |  | X |  |  | X |
| PBMC/plasma storage |  | X |  |  | X |
| Serum storage |  | X | X | X | X |
| Stipend | X | X | X | X | X |

**SCR = screening; ENR = enrollment; D1 = first dose day, MIDPT = midpoint visit with target date 21 days after first dose day , ENDPT = endpoint visit with target date 42 days after first dose day window period for each scheduled visit is ± 5 days of the target visit date**

*** Intensive PK sampling will be performed after 28 days of study drug on the 7 days/week arm. The window period for the intensive PK visit is ± 5 days.**

**†Hepatitis serological testing will be done at the screening visit and at the final endpoint visit for individuals who are susceptible to hepatitis B at baseline.**

**†HBV DNA testing will be performed for individuals who have isolated hepatitis B core antibody positivity (HBSAg negative, anti-HBs negative, anti-HBc positive)**

## Drug-Administration Flowchart

| **Procedure** | **Weekly follow-up (6 wks)** | | | | | | |
| --- | --- | --- | --- | --- | --- | --- | --- |
|  | **M** | **T** | **W** | **Th** | **F** | **S** | **Su** |
| **DOT (7 doses/week)** | **X** | **X** | **X** | **X** | **X** |  |  |
| **Phone mDOT (7 doses/week)** |  |  |  |  |  | **X** | **X** |
| **DOT (4 doses/week)** | **X** | **X** |  | **X** | **X** |  |  |
| **DOT (2 doses/week)** |  | **X** | **X** |  |  |  |  |

## Screening

Enrollment criteria will be evaluated at a screening visit. Screening evaluations will be used to determine the eligibility of each candidate for study enrollment. Eligible participants will be enrolled within 45 days. If a participant fails to enroll within 45 days of the screening visit and wishes to participate in the study, s/he may repeat the screening visit procedures.

Potential study participants will be screened by a research clinician and counselor. Participants will be told the purpose of the study and the reasons they have been approached for participation. A clinician will explain study procedures, including potential risks with study participation, and answer the participant’s questions. When a subject agrees to participate, s/he will sign an English consent form. The informed consent process is designed to maximize understanding of potential risks. The participant will have an opportunity to ask questions during the informed consent process. Subjects will be provided a copy of the consent form and a copy of the UCSF Experimental Subjects’ Bill of Rights. Participants will be informed that they can decline to participate without any change in their relationship with UCSF or the San Francisco Department of Public Health. They will also be informed that they can quit the study at any time. To protect the autonomy of volunteers, participants will not be given any academic credit or advantage for participation.

After written informed consent is obtained, participants will receive a symptom- directed medical and physical exam, a rapid HIV test, and screening safety laboratory tests (CBC, electrolyte panel, serum creatinine, liver function tests, total serum lipase, phosphorus, hepatitis B surface antigen, hepatitis B core antibody, hepatitis B surface antibody, urinalysis, and urine pregnancy test for women). Individuals who have isolated hepatitis B core antibody positivity will also have Hepatitis B DNA testing. Laboratory tests that fall outside the eligibility range may be repeated once within the screening period based on clinician discretion. All potential participants will receive standardized pre- and post-test risk reduction counseling and HIV testing by an FDA-approved commercially available whole blood rapid HIV test. Reactive rapid HIV test results must be confirmed with both another FDA-approved EIA and Western blot (WB) or immunofluorescent assay (IFA). Subjects will be assessed for eligibility by the research clinician and PI according to the eligibility criteria listed in sections 5.3 and 5.4. To ensure adequate hair at each sampling, participants will be advised to leave at least 2 cm of hair growth when getting their hair cut during this study.

If the participant is unable to complete all the screening procedures in one visit, they may return for one or more visits to complete these procedures.

| **Screening visit (up to 45 days prior to Enrollment visit)** | |
| --- | --- |
| Component | Procedure/Analysis |
| Educational/  Administrative | - Review educational materials/study flipchart with participant - Obtain written informed consent for screening - Assign Screening ID - Collect demographic information - Collect locator information - Assess eligibility - Provide reimbursement for visit - Schedule screen 2 and/or enrollment visit |
| Clinical | - Collect medical history - Record list of concomitant medications - Perform symptom-directed physical exam - Provide counseling   - HIV pre- and post-test counseling |
| Urine | - Collect urine sample   - Qualitative hCG   - Dipstick UA (and full UA with microscopy if greater than trace protein, glucose, heme, or leukocytes) |
| Blood | - Collect blood samples   - Complete blood count   - Serum chemistries   - Liver function tests   - Total serum lipase   - Hepatitis B testing   - HIV-1 rapid test (confirmatory testing as needed) |

## Enrollment

At the enrollment visit, the participant’s laboratory results will be reviewed with the study participant. Another rapid HIV test will be performed. All eligibility criteria will be reviewed and confirmed. If the participant is eligible, he or she will be enrolled and randomly assigned into one of 6 arms of this 3 condition cross-over study (see section 7.5). Each arm will be stratified by gender. The 4 doses/week group will occur on Monday, Tuesday, Thursday, and Friday, and the 2 doses/week will occur on Tuesdays and Wednesdays. Enrollments may be scheduled on any day of the week, but study drug initiation will occur at the beginning of the week (Monday for the 4 and 7 doses/week groups and Tuesday for the 2 doses/week group).

Blood, hair, and urine specimens will be collected at the enrollment visit. Participants will receive a detailed daily dosing schedule and visit calendar for all 3 periods of their study participation. The participants’ cell phone number, other phone numbers and a list of places where they might be located will be obtained along with a follow-up plan in the event that the participant is not at the designated meeting place.  A one-week supply of back-up medication will be provided in the event that participants are unable to be seen for directly observed doses. Study participants will be provided access to a 24 hour, 7 days/week emergency pager to discuss any potential study related adverse events with a study clinician.

| **Enrollment Visit** | |
| --- | --- |
| Component | Procedure/Analysis |
| Administrative | - Assign Participant ID or Screen failure ID - Review/update locator information - Provide lab results to participant - Confirm eligibility - Follow procedures for randomization assignment - Explain mDOT procedures - Confirm cell-phone number for doses observed by phone and reminder text messages - Provide dosing schedule and visit calendar to participant - Schedule next study visit with RA - Provide reimbursement for visit |
| Clinical | - Update medical history - Update list of concomitant medications - Perform full physical exam - Provide counseling   - HIV pre- and post-test counseling |
| Urine | - Collect urine sample   - Qualitative hCG   - Dipstick UA (and full UA with microscopy if greater than trace protein, glucose, heme, or leukocytes) |
| Blood | - Collect blood samples   - Complete blood count   - Serum chemistries   - Liver function tests   - Total serum lipase   - HIV-1 rapid test (confirmatory testing as needed)   - PBMC, plasma, and serum storage |
| Hair | - Complete standard hair collection questionnaire - Collect scalp hair sample - Self-collection of pubic hair sample (optional, opt-in procedure) |
| Study Product Supply | - Provide 1 week supply of back-up medication - Administer first dose (or schedule date of first dose) |

## Allocation Scheme

| **Sequence** | **N** | **Period A:**  **6 weeks** | **Break** | **Period B:**  **6 weeks** | **Break** | **Period C:**  **6 weeks** |
| --- | --- | --- | --- | --- | --- | --- |
| 1 | 4 (2M, 2F) | 7 doses/week |  | 4 doses/week |  | 2 doses/week |
| 2 | 4 (2M, 2F) | 7 doses/week |  | 2 doses/week |  | 4 doses/week |
| 3 | 4 (2M, 2F) | 4 doses/week |  | 7 doses/week |  | 2 doses/week |
| 4 | 4 (2M, 2F) | 4 doses/week |  | 2 doses/week |  | 7 doses/week |
| 5 | 4 (2M, 2F) | 2 doses/week |  | 7 doses/week |  | 4 doses/week |
| 6 | 4 (2M, 2F) | 2 doses/week |  | 4 doses/week |  | 7 doses/week |

## Randomization

The study statistician will randomize allocation sequences in two blocks of 6 within each gender stratum, (12 male and 12 female study participants). Each study participant will be randomly assigned to 1 of the 6 dosing sequences based on order of enrollment. Assignments will be concealed in sequentially numbered, opaque, sealed envelopes. Participants will be randomized to a sequence only after they have consented to participate, met eligibility criteria, and enrolled in the study.

## Follow-up Visits

Follow-up procedures at each visit are shown in Section 7.1. Adverse event assessments and symptom-directed physical exams will be performed by the study clinician every 3 weeks and additionally as needed during the study. The window period for scheduled study visits will be ± 5 days of the target visit date. Dosing will be continued at the assigned dosing frequency up until the endpoint visit occurs. No further doses for that dosing period will be given after the endpoint visit. HIV rapid testing and safety laboratory monitoring will also occur during these visits, and abnormal lab values will be followed clinically until stabilized. Adherence counseling will be performed at these visits. A hair sample will be collected on the last day of each treatment condition. Plasma samples for the intensive PK study will be collected after 4 weeks of dosing on the 7 doses/week condition.

## Directly Observed Visits and Phone Visits

Participants will receive modified directly observed dosing of oral TFV from a study staff according to the dosing schedules shown in section 7.2. Doses for all participants will be scheduled to occur at roughly the same time each day (e.g. 3pm). Ingestion of all Monday through Friday scheduled doses will be directly observed by study staff at an agreed-upon location on the UCSF campus, at the SFDPH research clinic, or at another predetermined location to maximize convenience for the participant. For weekends, holidays, and other foreseen-in-advance dates for self-dosing, study staff will provide pills to participants at the immediately preceding dose visit. Ingestion of unobserved doses will be confirmed via phone or text message mDOT. Participants will be asked to text or phone same-day confirmation of when they self-dose; if confirmation has not been received by a predetermined time, study staff will text and/or call asking for confirmation to help ensure that the doses are taken. Study staff will also send participants reminders as needed by email, text message, or telephone, for directly observed dose visits or to take back-up (emergency pack) medication if they miss a dosing visit. Dosing times will be recorded by the study staff for directly observed doses and telephone mDOT.  Study staff will confirm the dates and times of all self-administered doses at their next meeting with the subject.  Stipends will be provided at the completion of each dosing visit or clinical visit.

## Interim Contacts

Interim contacts and visits (those between regularly scheduled follow-up visits) may be performed at the participant request or as deemed necessary by the investigator or study staff at any time during the study. All interim contacts and visits will be documented in the participants’ study records.

## Criteria for Suspension of Study Product

Participants will be discontinued from receiving further study tablets for the following reasons:

- Confirmed HIV-1 infection
- Positive pregnancy test
- Positive hepatitis B surface antigen test or detectable Hepatitis B DNA
- Concurrent illness which, in the investigator’s opinion, may be exacerbated by continued product administration, requires treatment which is contraindicated by the protocol, jeopardizes further compliance or follow-up, or may confound the interpretation of adverse events
- Unacceptable toxicity, as defined in the toxicity management section of the protocol
- Participant request to discontinue for any reason
- Any clinical or social situation that, in the opinion of the investigator, jeopardizes compliance with the study product or confounds the interpretation of clinical adverse events

## Follow-up Procedures for Participants who Discontinue Study Product

Participants who discontinue study product will be encouraged to remain in the study if they are willing, for safety evaluations every 3 weeks according to the follow-up schedule in section 7.1. Since these participants will be off study drug, they will not complete directly observed or phone mDOT dosing visits. All study procedures described in the early study termination visit (in section 7.12) will be performed at the study drug discontinuation visit, including **hair** collection and PBMC**,** plasma, and serum collection. In addition, if a participant is advised to discontinue study drug by a clinician or the Principal Investigator during a dosing cycle due to an intercurrent illness, adverse event, or other circumstance, the participant may restart the unfinished dosing cycle at a later date if determined to be safe and appropriate by the Principal Investigator and allowable per protocol. Upon restarting the interrupted dosing cycle the participant would be required to complete the cycle in its entirety. This would include a start visit, midpoint visit, and an endpoint visit including all procedures and safety lab tests that are performed at each of these clinical visits. The process of repeating a dosing cycle will be at the discretion of the Principal Investigator. Participants who discontinue study drug and do not restart the dosing cycle will not participate in the intensive PK assessment after study drug discontinuation.

## Early Study Termination Visit

If a participant terminates study participation prior to the end of the study, he/she will be asked to have an Early Study Termination Visit and return any unused study product. At this visit, any evaluations showing abnormal results where there is a reasonable possibility of a causal relationship with the study drug should be repeated until the abnormality resolves or stabilizes. The following evaluations will be completed at the Early Study Termination Visit:

| **Early Study Termination Visit** | |
| --- | --- |
| Component | Procedure/Analysis |
| Administrative | - Provide lab results to participant - Provide reimbursement for visit |
| Clinical | - Review adverse events and intercurrent illnesses - Update list of concomitant medications - Perform symptom-directed PE - Provide counseling   - HIV pre- and post-test counseling |
| Urine | - Collect urine sample   - Qualitative hCG   - Dipstick UA (and full UA with microscopy if greater than trace protein, glucose, heme, or leukocytes) |
| Blood | - Collect blood samples   - Complete blood count   - Serum chemistries   - Liver function tests   - Total serum lipase   - HIV-1 rapid test (confirmatory testing as needed)   - Hepatitis B serological testing (if susceptible)   - **PBMC, plasma, and serum storage** |
| Hair | - Collect scalp hair sample (if on current regimen within the last 4 weeks) - Self-collection of pubic hair sample (optional, opt-in procedure) (if on current regimen within the last 4 weeks) |
|  |  |
| Study Product Supply | - Collect unused study medication from the participant |

In the event of early termination, participants may be replaced at the discretion of the PI and study statistician.

# DATA SOURCES

## Study Eligibility Assessment

Participants will undergo face-to-face interviews at the screening and enrollment study visits with trained staff. The screening interviews will obtain information on demographics, hair color, and study eligibility criteria.

## Clinical Assessment

A symptom directed physical exam will be performed at screening and a complete physical exam will be performed at enrollment by a trained clinician. Clinical symptoms will be systematically assessed every 3 weeks for the duration of the study. The severity of clinical symptoms will be scored using the established National Institutes of Allergy and Infectious Diseases (NIAID) Division of AIDS (DAIDS) toxicity scale. Additional data will be collected regarding concomitant medication use.

## Toxicity Monitoring

Blood and urine will be obtained at screening, enrollment, and then every 3 weeks while on study drug for toxicity monitoring. The severity of laboratory toxicity will be assessed and scored according to the DAIDS Toxicity scale (see section 9.6). Routine toxicity monitoring will include hematologic profile, chemistry profile (including sodium, potassium, chloride, total CO2 or bicarbonate, blood urea nitrogen, creatinine, random glucose, total bilirubin, alkaline phosphatase, phosphorus, AST, ALT, serum lipase), and urine dipstick.

## Monitoring for Pregnancy

Urine pregnancy testing will be performed for all female study participants at screening, enrollment, at every 3 week follow-up visit, and at the early study termination visit.

## HIV-1 Testing

HIV-1 serological testing will be performed using an FDA-approved whole-blood EIA rapid test at screening, enrollment, and every 3 weeks by trained study staff (except during breaks). Additional HIV serologic testing will be performed at interim visits on participant request. Non-reactive tests will be reported as non-reactive. Participants found to have reactive antibody tests will receive confirmatory testing with both a second FDA-approved EIA and WB or IFA.

## Hair Specimens

Hair specimens will be collected at enrollment and on the last day of each 6-week dosing period. A standard questionnaire will be completed at each hair collection visit to record information about hair treatments and type and number of hair samples collected. Hair specimens will be used to determine tenofovir hair concentrations under each dosing condition. Hair collection will be conducted by study staff trained on proper hair collection technique and will be stored at room temperature in aluminum foil in a secured, dark location until analyzed. Scalp hair will be collected on all study participants.

Participants will also be asked to provide a pubic hair specimen on an optional, opt-in basis using a self-collection technique. Pubic hair TFV level testing could be useful in certain parts of the world (e.g. sub-Saharan Africa) where men and women shave their heads or in individuals who dye their hair. Evaluating the correlation between scalp and pubic hair TFV concentrations will help determine whether pubic hair TFV testing could be a possible substitute for scalp hair in situations where scalp hair is unavailable. Those participants who agree to hair collection would be shown a sample of 200 pubic hairs and the proper technique for self-collection, including placement of a sticker on the distal end of the hair, cutting hair as close to the skin as possible, and placing hair in aluminum foil for storage.

All hair and blood samples (see sections 8.6 – 8.8) will be run in batch after all samples have been collected. The hair and pharmacology laboratory staff will remain blinded to the dosing condition until all testing is completed.

## Intensive PK Specimens

After 4 weeks of dosing on the 100% adherence condition, all participants will undergo a 24 hour intensive-PK study protocol (requiring an approximately 25-hour-long hospital stay) to evaluate steady-state effects of TFV in plasma. This visit will take place at the Clinical Resource Center (CRC) associated with UCSF/Moffitt Hospital.

The participant’s usual diet will be ascertained by phone prior to the PK visit, and simulation of the usual diet will be undertaken in the CRC during PK sampling. During the week before the PK study visit, the participant will complete questionnaires ascertaining current medication and substance use patterns, current dietary patterns, and current menstrual, contraceptive, and obstetric history, if applicable. An initial blood level will be drawn (“0” timepoint) and consumption of one dose of oral tenofovir will be witnessed. A series of blood samples will then be collected into EDTA tubes at 0, 0.25, 0.5, 1, 1.5, 2, 3, 4, 6, 8, 10, 12, 16, and 24 hours post-dose after witnessed dosing.

## PBMC Specimens

Peripheral blood mononuclear cells (PBMCs) will be collected at enrollment and at the 6 week visits for each dosing condition. PBMCs will be used to determine association between dose and intracellular tenofovir diphosphate (TFV-DP) levels, correlating TFV hair levels with TFV-DP levels, performing pharmacogenomic studies on the influence of single-nucleotide polymorphisms on TFV levels, and conducting in vitro challenge experiments to determine minimum inhibitory TFV dose required to inhibit viral replication (section 8.9).

## HIV-1 Cultures of PBMCs in vitro

Surrogate markers of the protective effects of tenofovir are needed to facilitate future optimization of chemoprophylaxis dose.  We hypothesize that drug dose, hair drug levels, or intracellular drug levels in this study will correlate with susceptibility of blood cells to HIV-1 measured ex vivo.  Peripheral blood mononuclear cells will be processed at the UCSF Laboratory of Clinical Virology using methods that yield specimens that are suitable for viral culture (i.e.: separation over ficoll hypaque).  These cells will be stimulated in vitro possibly using PHA or anti-CD3 antibodies in IL-2 containing media.  If required to allow viral replication, CD8 cells will be depleted from the cell specimens using anti-CD8 antibodies.  The stimulated cells will be challenged with a dilution series of a standardized stock of HIV-1 to determine the infectious dose sufficient to infect 50% of the culture wells (TCID50).  Viral challenges will occur within 24 hours of cell culture to minimize the decay of drug concentrations inside the cells.  Standard approaches to evaluate viral replication will be used, and may include measurement of p24 antigen in the culture supernatant or detection of markers expressed by viral test vectors, such as luciferase or GFP.  All work will be performed under a current UCSF approved BUA in the laboratory.  Participants will not be exposed to HIV-1 during these procedures; rather, the blood cells obtained by phlebotomy will be analyzed ex vivo in the laboratory.  No participant identifiers will be shared with the laboratory.  The viral culture assays are not validated for clinical use so individual results will not be shared with the study participants.  Values obtained using cells derived during therapy will be compared with values obtained using cells prior to therapy to estimate the fold change in TCID50 associated with every dosage level and intracellular TNF-DP level.

## Additional Cryopreservation of Blood Specimens

At visits where PBMCs are collected, leftover plasma specimens will be cryopreserved for future testing. These specimens may be used for (1) analysis of plasma TDF levels, (2) analysis of lipids and other parameters of lipid or glucose metabolism, (3) testing for additional markers of renal function, such as cystatin C, and (4) testing of markers of bone turnover. In addition, serum will be storage at visits where blood is drawn for possible future testing of novel markers of renal function (Cystatin C) and markers of bone turnover.

# ADVERSE EVENTS

An adverse event (AE) is any untoward medical occurrence in a clinical research participant enrolled in a clinical trial, regardless of causality assessment. An AE can be any unfavorable or unintended sign, symptom, or disease temporally associated with the use of an investigational product or study participation, whether or not considered related to the product or study participation. Pre-existing conditions, which increase in frequency or severity or change in nature during or as a consequence of use of a drug in human clinical trials, will also be considered as adverse events. AEs may also include pre- or post-treatment complications that occur as a result of protocol-mandated procedures. AE assessment will begin at the time of randomization, and conditions noted prior to randomization will be noted as pre-existing conditions.

## Assessment of Adverse Events

Study participants will be provided instructions for contacting the study site to report any untoward medical occurrences they may experience, except for possible life-threatening events, for which they will be instructed to seek immediate emergency care. With appropriate permission of the participant, and whenever possible, records from all non-study medical providers related to untoward medical occurrences will be requested and required data elements will be recorded on study CRFs and/or in the participant’s medical chart. All AEs will be followed clinically, until the AE resolves or stabilizes as per the appropriate toxicity algorithm.

## Serious Adverse Event

Serious adverse events (SAEs) will be defined per CFR 312.32 guidelines, as AEs occurring at any dose that:

- Result in death
- Are life-threatening adverse events
- Require inpatient hospitalization or prolongation of existing hospitalization
- Result in persistent or significant disability/incapacity, or
- Are congenital anomalies/birth defects

Important medical events that may not result in death, be life-threatening, or require hospitalization may be considered serious when, based upon appropriate medical judgment, they may jeopardize the participant or require medical or surgical intervention to prevent one of the outcomes listed above.

## Serious Adverse Event Reporting Requirements

All serious adverse events will be reported to the principal investigator within 72 hours of knowledge of the SAE. In addition, all grade 3 or higher adverse events and all creatinine elevations ≥ 0.5 mg/dL over baseline will be reported to the medical officer within this timeframe (72 hours of knowledge). These events may be reported via telephone or email. For hospitalizations, fatal, or life-threatening events, copies of hospital reports, autopsy reports, and other documents will be requested. The investigator will identify all therapeutic measures necessary for resolution of these events. Follow-up of these events will continue until the medical officer determines that the condition has stabilized or resolved.

This study will follow UCSF CHR reporting requirements for adverse events. All serious or unexpected adverse events that are definitely, probably, or possibly related to study participation will be reported to the UCSF IRB within 10 working days of site awareness. The NIMH Project Officer will be provided copies of these reports and informed of any actions taken by the IRB as a result of such events. Finally, Gilead will be notified of all SAEs that may be related to study drug (probably not related, possibly related, probably related, and definitely related).

## Recording of Clinical and Laboratory AEs

All clinical AEs will be assessed by a licensed clinician and recorded on the study Illness/AE log CRF. Laboratory values will be captured and graded in a laboratory database. The following laboratory abnormalities will be recorded on the AE CRFs: 1) grade 2 or higher laboratory AEs on the DAIDS toxicity table, 2) laboratory AEs that result in study drug interruption or discontinuation, require a concomitant medication, or are associated with symptoms, or 3) laboratory AEs that the investigator or clinician feels are clinically significant. If the laboratory abnormality is part of a syndrome, the overall syndrome or diagnosis will be recorded.

## Adverse Event Relationship to Study Product

The relationship of all AEs to study product will be assessed per the Manual for Expedited Reporting of Adverse Events to DAIDS (dated 6 May 2004), the package insert and investigator’s brochure for Viread, and clinical judgment of the investigator. The relationship categories that will be used for this study are:

- Definitely related: adverse event and administration of study agent are related in time, and a direct association can be demonstrated with the study agent
- Probably related: adverse event and administration of study agent are reasonably related in time, and the adverse event is more likely explained by the study agent than by other causes
- Possibly related: adverse event and administration of study agent are reasonably related in time, and the adverse event can be explained equally well by causes other than the study agent.
- Probably not related: a potential relationship between administration of study product and adverse event could exist, but is unlikely, and the adverse event is most likely explained by causes other than the study agent
- Not related: the adverse event is clearly explained by another cause unrelated to administration of the study product. Reportable events must have documentation to support the determination of “not related.”

## Grading Severity of Adverse Events

All AEs and abnormal laboratory values will be graded using the NIAID DAIDS AE Grading Table (Appendix 2). For laboratory AEs where there is a gap between grades (i.e. for creatinine grade 1 AE = 1.43 – 1.69 mg/dL, and grade 2 range = 1.82 – 2.34 mg/dL for ULN = 1.3), the higher grade will start at the upper limit of the lower grade (grade 2 creatinine elevation will be begin at 1.70 mg/dL).

## Toxicity Management

In general, the site investigator has the discretion to hold study product at any time if he feels that continued product use would be harmful to the participant or interfere with treatment deemed clinically necessary according to the judgment of the investigator. Clinical or laboratory abnormalities that require follow up will be documented, and the research associate or clinician will contact the participant to schedule an interim visit for follow-up and/or repeat laboratory testing. All participants reporting an untoward medical occurrence will be followed clinically until the occurrence resolves (returns to baseline grade, defined as grade at enrollment) or stabilizes.

Grade 1 and 2 Laboratory Abnormality or Clinical Event (excluding creatinine elevations)

Continue study product at the discretion of the site investigator.

Grade 3 Laboratory Abnormality or Clinical Event (excluding creatinine elevations)

Study medication may be continued at the discretion of the site investigator if a grade 3 toxicity is considered to be unrelated or probably not related to the study medication. Study medication will be temporarily withheld if Grade 3 toxicity is considered to be possibly related, probably related, or definitely related to the study medication. Laboratory toxicity will be promptly confirmed by repeating the test on an additional specimen, preferably within 7 days. After a Grade 3 toxicity returns to Grade 1, the participant can be reintroduced to medication. If a Grade 3 toxicity recurs and is considered to be probably or definitely related to study medication, the study medication will be permanently discontinued.

Grade 4 Laboratory Abnormality or Clinical Event (excluding creatinine elevations)

For all Grade 4 laboratory-identified or clinical toxicities, the study medication will be withheld. Laboratory toxicity will be promptly confirmed by repeating the test on an additional specimen, preferably within 7 days. If a grade 4 laboratory toxicity is not confirmed by repeat testing, it should be managed per algorithm for the new toxicity grade. Participants with grade 4 AEs will be followed until the event resolves to baseline or stabilizes. If a grade 4 toxicity is considered to be unrelated or probably not related to study product and resolves to baseline, study drug may be restarted at the discretion of the site investigator. If the toxicity recurs to grade 3 or higher after study medication is restarted and is considered to be probably or definitely related to study medication, the study medication will be permanently discontinued.

### Creatinine Elevations

For creatinine elevations ≥ 0.5 mg/dL over baseline creatinine (at enrollment), serum creatinine will be repeated as soon as possible, preferably within 7 days, and the protocol chair or designated medical officer will be notified within 72 hours. Study product will be held for confirmed creatinine elevations ≥ 0.5 mg/dL over baseline or creatinine clearance < 50 mL/min. Study product should be held until creatinine returns to within 0.3 mg/dL of enrollment level, at which point the participant may be rechallenged with study drug after consultation with the protocol chair. If serum creatinine rises again to ≥ 0.5 mg/dL over baseline or creatinine clearance drops to below 50 mL/min when drug is restarted, study product should be permanently discontinued, and the participant will be monitored until level returns to baseline (enrollment value) or stabilizes.

## Management of Intercurrent Medications

Participants who begin taking any medication during the trial that is listed as an exclusionary medication at screening will temporarily discontinue study drug. Study drug may be resumed if no exclusionary medication has been taken in the last 4 weeks, serum creatinine is within 0.3 mg/dL of enrollment level, and a rapid test for HIV antibodies is negative.

## HIV and Hepatitis B

Participants who are identified as infected with HIV and/or hepatitis B will permanently discontinue study drug and be provided linkages to care.

## Clinical Management of Pregnancy

All female study participants are required to be using an effective method of contraception according to section 5.3, and intending to use this method for the duration of study participation. Study staff will provide contraceptive counseling to enrolled female participants as needed throughout the duration of study participation and will facilitate access to contraceptive services through referrals to local service providers. Study staff will also provide all participants with male and/or female condoms and counseling on use of condoms during study participation.

Pregnancy testing will be performed at all study visits for female participants, and participants will be encouraged to report all signs or symptoms of pregnancy to study staff. Participants who become pregnant during the course of the study will permanently discontinue study drug. The principal investigator or clinician will counsel any participant who becomes pregnant regarding possible risks to the fetus and refer the participant to all applicable services; however, sites will not be responsible for paying for pregnancy-related care.

Participants who are pregnant at the termination visit will continue to be followed through phone contact with study staff until the pregnancy outcome is ascertained (or the investigator team determines that the pregnancy outcome cannot be ascertained). Pregnancy outcomes will be reported on relevant case report forms.

## Social AEs

Participation in the study could lead to social harms that may include loss of privacy or stigmatization. Information regarding social harms will be collected during follow-up visits and will be recorded in log form. All social harms will be brought to the attention of the medical officer who will determine appropriate follow-up.

## Review of AEs

The principal investigator will serve as the medical officer for this trial and review all laboratory and clinical adverse events weekly. As described in section 9.3, all SAEs, grade 3 or higher AEs, and creatinine elevations ≥ 0.5 mg/dL over baseline will be reported to the medical officer within 72 hours. The principal investigator will meet with appropriate members of the investigator team on a monthly basis to discuss enrollment, retention, and safety of study participants.

# DATA MANAGEMENT AND STATISTICAL CONSIDERATIONS

This is a three-period, open label, single-site crossover study. All enrolled participants will undergo 3 dosing regimens. The sequence of these dosing regimens will be randomly assigned. The total length of follow-up is approximately 36 weeks (18 weeks on study drug).

## Study Primary Endpoint and Predictor Variables

Consistent with the primary study objectives, the following primary endpoint will be assessed:

- Tenofovir hair concentration: Tenofovir hair levels will be determined from a specimen containing 150-200 hairs collected at enrollment and on the last day of each period. For each participant, absolute hair levels and ratios of hair levels between conditions (7 vs. 4 doses/week, 4 vs. 2 doses/week, and 7 vs.2 dose/week) will be determined.

The following will be primary predictor variables:

- Dosing condition: The dosing condition will be examined as an ordinal variable: 2, 4, and 7 doses/week. We will also use actual percentage of total doses (by directly observed recorded doses and self-administered recorded doses) taken over the 6 week period as a continuous predictor of hair levels.
- PK parameters: The primary predictor variable of total exposure (PK contribution) will be oral clearance (CL/F). This variable is estimated by the equation “dose/AUC,” where AUC is the area under the plasma-concentration time curve for each intensive PK sampling procedure. Other parameters of drug exposure that will be evaluated as predictor variables include the concentration at the end of the dosing interval (trough or C_min_), peak plasma level (C_max_), and AUC alone. These PK variables will be calculated using non-compartmental methods.

## Study Hypotheses

The study hypotheses for the primary objectives are:

- Tenofovir hair levels will correlate highly with frequency of tenofovir dosing. We expect hair levels from the 7 doses/week condition will exceed hair levels for the 2 doses/week condition. In addition, we expect that few doses from the 4 doses/week condition will exceed hair levels observed in the 7 doses/week condition, and few hair levels from the 2 doses/week condition will exceed hair levels observed in the 4 doses/week condition.
- Decreased oral clearance (CL/F) and higher AUC, C_min_, and C_max_ values will be associated with higher absolute TFV hair levels and an increased ratio of TFV hair concentrations between doses.

## Biostatistical Methods

Analyses for all aims will include initial examination of summary statistics and graphical summaries of data, checking of model assumptions, and careful examination of estimates and their confidence intervals rather than exclusive attention to p-values. When parametric assumptions are violated, nonparametric alternatives will be evaluated. Because multivariate modeling and controlling for potential confounders are difficult with nonparametric methods, bootstrapping strategies will also be considered for obtaining valid p-values and confidence intervals for parametric models whose assumptions are violated. This approach also has the advantage of providing estimates and confidence intervals, which are often unavailable with nonparametric methods. Over-interpretation of isolated or implausible findings of nominal statistical significance will be avoided by examination of corresponding estimates and confidence intervals and of related results while taking account of the scientific context and relationships among analyses (rather than by formal multiple comparisons adjustments).

The primary parameters of interest in this study will be TFV hair concentration under each dosing condition. We will determine mean, median, and 95% confidence intervals for this parameter for each dosing condition being tested. We will then estimate ratios of TFV levels in hair of different pair-wise combinations of dosing conditions and determine mean, geometric mean, median, and 95% confidence intervals for these ratios. We will also assess the ability of hair levels to discriminate between conditions, in particular noting how often any level under 4 day/week dosing exceeds any level observed under 100% adherence, and how often any level under 2 day/week dosing exceeds any level observed under 4 day/week or 7 day/week dosing.

We will first use scatterplots with LOWESS smooths to examine the relationships between TFV hair concentration and PK parameters calculated from the intensive pharmacokinetic studies, as well as the relationships between dose and hair concentration stratified by PK values. These PK parameters include clearance (CL/F) as estimated by dose/AUC, C_min_ or trough level, C_max_ or peak level, t_max_ or time to reach peak levels, and area-under-the-curve (AUC). Linear and possibly non-linear mixed models for TFV levels in hair will be used to assess these relationships. Random effects for participant will be used to account for within-subject correlation and thus capture the efficiency gains afforded by the crossover design. Outcomes will be normalized as necessary to meet model assumptions, and bootstrap confidence intervals computed as a sensitivity check. With log-transformed TFV hair levels as the outcome, effect estimates for both dose and PK variables will be interpretable in terms of percentage effects on TFV hair concentration. We will first estimate the overall effect of PK variables on TFV hair levels, carefully considering non-linear relationships and if necessary using non-linear mixed models. For example, we expect that lower clearance and higher AUCs will be associated with higher hair levels. In addition, we will examine modification of the associations between dose and hair concentrations by PK variables; in particular, we hypothesize that hair concentration will increase more rapidly across the 3 dose levels in participants with lower clearance and higher AUC.

## Sample Size

This study will provide the first information on how frequency of dosing influences hair levels of tenofovir, so there is no preliminary data on the person-to-person variability in hair level ratios between conditions. Primary interest will focus on describing typical ratios and their variability, as well as on a preliminary assessment of how well hair levels can distinguish 100% adherence from partial adherence. With 24 subjects and a mix of both sexes, little or no overlap between the distributions of hair levels under different conditions would strongly suggest substantial potential utility of hair levels for explaining breakthrough HIV infections in prevention studies.

There is no preliminary data on the within-person correlation of hair concentration levels, on which to base formal power calculations for the mixed model. However, for hair concentration to be a useful surrogate, it will need to be reliable – that is, highly correlated within-subjects – and very strongly associated with PK variables and dose. The proposed mixed models will provide preliminary estimates of the within-subject correlation of TFV hair levels, as well as estimates of the strength of the associations between hair concentration, dose, and PK variables.

## Data Management

Management and cleaning of data will be performed by the data management teams at the San Francisco Department of Public Health and UCSF. Drug level measurements will be transmitted as Excel files from the UCSF hair lab to the data manager and imported into a SAS database for analysis.

# HUMAN SUBJECTS CONSIDERATIONS

The investigators will make efforts to minimize risks to human participants. Volunteers and study staff members will take part in a thorough informed consent process. Before beginning the study, the investigators will have obtained IRB approval.

## Institutional Review Boards

This study will be reviewed by the University of California, San Francisco Committee on Human Research (CHR). Prior to implementation of the study, the protocol and consent forms will be approved by this IRB. All recruitment and retention materials will be approved by the CHR prior to use. Any amendments to the protocol, informed consents, or other study-related documents will also be approved by the CHR prior to implementation.

## Informed Consent

During the screening process for this study, potential study participants will be told the purpose of the study and the reasons they have been approached for participation. A clinician will explain study procedures, including potential risks with study participation. When a subject agrees to participate, s/he will sign an English consent form, as all participants will be English speaking. A comprehension test will be used to assess participants’ comprehension of the informed consent document. Participants who are unable to demonstrate adequate understanding of key concepts after educational efforts will not be enrolled in the study. Participants will be provided a copy of the consent form as well as a copy of the UCSF Experimental Subjects’ Bill of Rights. Participants will be informed that they can decline to participate without any change in their relationship with UCSF or the San Francisco Department of Public Health. They will also be informed that they can quit the study at any time. To protect the autonomy of volunteers, participants will not be given any academic credit or advantage for participation.

## Participant Confidentiality

Participation in research may result in a loss of confidentiality. To minimize this risk, all project staff will be trained in procedures for maintaining confidentiality. All data will be coded by a subject number. Data will be kept in double-locked cabinets, and any forms with identifying information will be stored separately from the remaining study data. Research records will be kept confidential to the level allowed by law. All study staff will be trained on procedures for maintaining privacy and will sign an oath of confidentiality. Any information provided by study participants will not be revealed to any outside parties unless written consent is provided by the study participant. Participant visits will be conducted in private locations agreed upon by the study participant and staff. UCSF faculty will not be told which students are participating in the study.

All local databases will be secured with password-protected access systems. Forms, lists, log books, appointment books, and any other listings that link participant ID numbers to other identifying information will be stored in a separate, locked file in an area with limited access.

## Risks

Anticipated risks include the following:

1. *Phlebotomy*: Risks of minor injury, such as bruising or infection with venipuncture are unlikely.
2. Risk of loss of privacy or confidentiality
3. Hair collection: temporary change in appearance of hair.
4. *Study drug and toxicities.*  Risks and side effects related to the study drug TDF include the following:

- Gastrointestinal intolerance (such as nausea, abdominal pain, diarrhea, or vomiting)
- Flatulence
- Headache
- Rash.
- Redistribution/accumulation of body fat. This has been observed in HIV-infected patients receiving combination antiretroviral therapy. The mechanism and long-term consequences of these events are currently unknown. A causal relationship has not been established. These effects are not expected in this study involving a brief exposure to antiretroviral drug.

Rare but serious side effects include:

- Lactic acidosis/severe hepatomegaly with steatosis
- Renal impairment, including cases of acute renal failure and Fanconi’s syndrome (renal tubular injury with severe hypophosphatemia)
- Increase in bone metabolism leading to osteopenia or osteomalacia
- Hypersensitivity reaction

## Benefits

Participation in this study will likely have no direct benefit to volunteers. Volunteers will receive free medical and laboratory monitoring (including serology, blood count, liver and kidney function tests) which may present the opportunity for early diagnosis and treatment of undiagnosed disease. The participant may appreciate the opportunity to contribute to the body of knowledge in the fields of HIV prevention and pharmacokinetics.

## Access to Clinician

Study participants will be provided access to a 24 hour, 7 days/week emergency pager to discuss any potential study related adverse events with a study clinician.

## Special Populations

This section outlines considerations made for the inclusion or exclusion of special populations in this study.

### Pregnant Women

Participants who test positive for pregnancy at screening or enrollment visits will not be eligible to participate in this study. A urine pregnancy test will be performed on all women at all clinic visits (every 3 weeks while on study drug), and participants who test positive will be taken off product. During the informed consent process, women will be informed that the effects of oral tenofovir disoproxil fumarate on a developing human fetus are unknown.

Oral TDF is classified by the FDA as a Pregnancy Category B drug. Animal studies have failed to demonstrate a risk to the fetus, but there are no adequate and well-controlled studies in pregnant women that have been completed to date.

All potential participants will be required by the eligibility criteria to be currently using a reliable method of contraception, such as condoms (with or without a spermicide), diaphragm or cervical cap with spermicide, intrauterine device, hormonal contraception, or successful vasectomy in the male partner.

### Children

Oral TDF is not currently approved for children under 18 years old. This study does not plan to enroll children under 18 years old. Individuals at least 18 years old will be eligible for enrollment.

## Incentives

Participants will be paid for their time and effort in this study. Stipends will be paid to participants based on the number of scheduled doses and clinical visits completed.

## Treatment for Injury

Participants will be asked to inform the study staff if they feel they have been injured because of taking part in the study. Injury may also be identified during laboratory testing, medical histories, and physical examinations. These injuries will be reported as AEs. Treatment will be available at UCSF, through the San Francisco Department of Public Health, or through linkages to local providers.

## Data and Safety Monitoring

This clinical study is determined to be of moderate risk to subject participants. This is an open label study, and tenofovir has been demonstrated to be safe for treatment of HIV infection and is approved by the US FDA for this indication. In addition, there were no differences in clinical or laboratory adverse events reported between tenofovir vs. placebo in a double-blinded placebo-controlled trial of tenofovir chemoprophylaxis trial in 936 HIV-negative women in Africa. Toxicities will be minimized by the limited duration of exposure to tenofovir (18 weeks total) and frequent safety monitoring for adverse events. Safety monitoring will include symptom directed physical exams, assessment of adverse events, laboratory monitoring, and rapid HIV testing every 3 weeks. Adverse events will be graded based on the Division of AIDS Grading table or as described in section 9.6. All SAEs, grade 3 or higher AEs, and all creatinine elevations ≥ 0.5 mg/dL over baseline will be reported to the medical officer within 72 hours. Unexpected or serious adverse events that occur during the course of the study will be reported to the Committee on Human Research (Institutional Review Board) at the University of California (UCSF) in accordance with current University guidelines for reporting adverse events. Patients will have a 24-hour contact number to report any potential adverse events that occur in between scheduled study visits. The principal investigator will review all laboratory and clinical adverse events weekly. In addition, appropriate members of the investigator team will meet on a monthly basis to discuss enrollment, retention, and safety of study participants.

## Study Discontinuation

This study may be paused or stopped at any time by the National Institutes of Mental Health, the Office for Human Research Protections (OHRP), the UCSF IRB, or principal investigator of the study.

## Biohazard Containment

Universal precautions will be practiced under the assumption that any human specimen could be infected with HIV-1 or other blood-borne pathogens. Biohazardous wastes are discarded in marked bins and decontaminated by incineration.

# ADMINISTRATIVE PROCEDURES

Study case report forms will be developed by the investigator team. The investigator will maintain, and store securely, complete, accurate, and current study records throughout the study. In accordance with US regulations, the investigator will retain all study records on site for at least two years after study closure. Applicable records include source documents, case report forms, informed consent forms, and notations of all contacts with the participant.

# REFERENCES

1. Joint United Nations Programme on AIDS (UNAIDS) and World Health Organization (WHO). *AIDS epidemic update* 2008.

2. Youle M, Wainberg MA. Pre-exposure chemoprophylaxis (PREP) as an HIV prevention strategy. J Int Assoc Physicians AID Care. 2003;2:102-105.

3. Liu AY, Grant RM, Buchbinder SP. Preexposure prophylaxis for HIV: unproven promise and potential pitfalls. JAMA. Aug 16 2006;296(7):863-865.

4. Cohen MS, Gay C, Kashuba AD, Blower S, Paxton L. Narrative review: antiretroviral therapy to prevent the sexual transmission of HIV-1. Ann Intern Med. Apr 17 2007;146(8):591-601.

5. Grant RM, Buchbinder S, Cates W, Jr., et al. AIDS. Promote HIV chemoprophylaxis research, don't prevent it. Science. Sep 30 2005;309(5744):2170-2171.

6. Paxton LA, Hope T, Jaffe HW. Pre-exposure prophylaxis for HIV infection: what if it works? Lancet. Jul 7 2007;370(9581):89-93.

7. Katzung B. Basic and Clinical Pharmacology. 10th ed; 2007.

8. Krishnamurti U, Steffes MW. Glycohemoglobin: a primary predictor of the development or reversal of complications of diabetes mellitus. Clin Chem. 2001;47(7):1157-1165.

9. Home P, Chacra A, Chan J, Emslie-Smith A, Sorensen L, Crombrugge PV. Considerations on blood glucose management in Type 2 diabetes mellitus. Diabetes Metab Res Rev. Jul-Aug 2002;18(4):273-285.

10. Gandhi M, Greenblatt RM. Hair it is: the long and short of monitoring antiretroviral treatment. Ann Intern Med. Oct 15 2002;137(8):696-697.

11. Nakahara Y. Hair analysis for abused and therapeutic drugs. J. Chromatogr. B. Biomed. Sci. App. 1999;733:161-180.

12. Alexander J, Reistad R, Hegstad S, et al. Biomarkers of exposure to heterocyclic amines: approaches to improve the exposure assessment. Food Chem Toxicol. Aug 2002;40(8):1131-1137.

13. Cone E. Mechanisms of drug incorporation into hair. Ther Drug Monit. 1996;18:438-443.

14. Pepin G, Gaillard Y. Concordance between self-reported drug use and findings in hair about cocaine and heroin. Forensic Sci Int. Jan 17 1997;84(1-3):37-41.

15. Kelly RC, Mieczkowski T, Sweeney SA, Bourland JA. Hair analysis for drugs of abuse. Hair color and race differentials or systematic differences in drug preferences? Forensic Sci Int. Jan 10 2000;107(1-3):63-86.

16. Kosuge K, Uematsu T, Araki SI, Matsuno H, Ohashi K, Nakashima M. Comparative dispositions of ofloxacin in human head, axillary, and pubic hairs. Antimicrob Agents Chemother. May 1998;42(5):1298-1302.

17. Cassani M, Da Re N, Giuliani L, Sesana F. Experience with hair testing in the clinical biochemistry laboratory of Ca' Granda Niguarda Hospital, Milan, Italy. Forensic Sci Int. Jan 17 1997;84(1-3):17-24.

18. Gandhi M et al. Protease Inhibitor Levels in Hair Samples Strongly Predict Virologic Responses to HIV Treatment. AIDS. In press.

19. Mitty JA, Huang D, Loewenthal HG, MacLeod C, Thompson L, Bazerman LB. Modified directly observed therapy: sustained self-reported adherence and HIV health status. AIDS Patient Care STDS. Dec 2007;21(12):897-899.

20. Goggin K, Liston RJ, Mitty JA. Modified directly observed therapy for antiretroviral therapy: a primer from the field. Public Health Rep. Jul-Aug 2007;122(4):472-481.

21. Ma M, Brown BR, Coleman M, Kibler JL, Loewenthal H, Mitty JA. The feasibility of modified directly observed therapy for HIV-seropositive African American substance users. AIDS Patient Care STDS. Feb 2008;22(2):139-146.

22. Gilead Sciences. Viread (tenofovir disoproxil fumarate) tablets. Package Insert. November 2008.

# LIST OF APPENDICES

Appendix 1: Viread Package Insert

Appendix 2: Division of AIDS Table for Grading the Severity of Adult and Pediatric Adverse

Events (December, 2004)
